# Enrichment of the Conserved RY-Repeat Cis-Element in Maize upon *Ustilago maydis* Infection and Its Interaction with the Candidate Secreted Protein UMAG_06102

**DOI:** 10.3390/plants15203215

**Published:** 2026-10-20

**Authors:** Xinsen Ruan, Xiucai Pan, Qing Wang, Xiquan Gao

**Affiliations:** 1 State Key Laboratory for Crop Genetics and Germplasm Enhancement, Nanjing Agricultural University, Nanjing 210095, China; xsence@126.com (X.R.); qingwang@njau.edu.cn (Q.W.); 2 Jiangsu Collaborative Innovation Center for Modern Crop Production, Nanjing Agricultural University, Nanjing 210095, China; 3 College of Agriculture, Nanjing Agricultural University, Nanjing 210095, China; 4 Hubei Key Laboratory of Food Crop Germplasm and Genetic Improvement, Food Crops Institute, Hubei Academy of Agricultural Sciences, Wuhan 430064, China; pxcaice@163.com; 5 Xiangyang Academy of Agricultural Sciences, Xiangyang 441106, China

**Keywords:** maize, RY-repeat, candidate secreted protein, tumor formation, *Ustilago maydis*, *ZmSWEET*

## Abstract

*Ustilago maydis* causes corn smut diseases by forming tumors on maize. Effectors of *U. maydis* could regulate host gene transcription. However, there is remaining uncertainty about the mechanisms underlying the regulation of transcription reprogramming of maize genes by *U. maydis* to facilitate disease progression. Here, we conducted systemic enrichment analysis of cis-elements in maize genes induced by infection with *U. maydis*. Yeast library screening and gene expression analysis were used to identify candidate proteins that could interact with the RY-repeat-containing promoter regions. Knocking out and genetic variation analysis were also deployed to study the role of fungal protein binding to RY-repeat during *U. maydis* infection. The conserved plant cis-element RY-repeat in maize was enriched in response to *U. maydis*. UMAG_06102, a candidate secreted protein of *U. maydis*, showed a Y1H-detected interaction with the RY-repeat-containing region of *ZmSWEET11* promoter in a yeast-one-hybrid system. Knocking out *UMAG_06102* was associated with the reduced expression of *ZmSWEET11* and a decrease in virulence. Moreover, the genetic variation of the RY-repeat of *ZmSWEET11* was likely associated with the disease incidence of *U. maydis* on maize. Our results suggested that *U. maydis* could influence the host gene expression through the enrichment of the RY-repeat of *ZmSWEET11*, thus impacting tumor formation on host tissues.

## 1. Introduction

The basidiomycete fungus *U.* is one of the biotrophic pathogens which causes corn smut on maize, typically characterized by tumors on aerial organs. The life cycle of *U. maydis* consists of haploid and dikaryotic forms, which undergo nonpathogenic asexual reproduction or pathogenic sexual cycle, respectively [1]. *U. maydis* usually rely on living plant tissue via biotrophic parasitism and the sexual cycle, not killing host cells but interacting with the living host. Thus, *U. maydis* withdraws nutrients from the host to support fungal growth and development, and to establish its successful colonization of the maize host [2,3].

It has been reported that the glycol-metabolism could benefit the growth and pathogenicity of *U. maydis* during infection [4]. Induction of sucrose degradation and elevated levels of free hexose were observed in infected leaves [5]. Moreover, a series of sugar transporters from *U. maydis* have been reported to be involved in the interaction between *U. maydis* and maize. For instance, the sucrose-specific transporter Srt1 is essential for successful colonization within the host [6,7], and Hxt1, a high-affinity glucose transporter, might act as the glucose sensor to modulate pathogenicity [8]. Moreover, ZmSUT1, an H+ sucrose transporter, was also reported to contribute to the accumulation of sugar, which provides nutrients for *U. maydis* [7]. Furthermore, the expression level of the plant sugar transporters *SWEET* (*Sugar will eventually be exported transporter*) was highly correlated with the sugar accumulation within the tumor site [9]. The *SWEET* gene family encodes uniporters located on the plasma membrane, which transport sugars across cell membranes along with a concentration gradient [10]. SWEET proteins exist in various species, including bacteria, fungi, plants, and animals, containing a highly conserved MtN3/Saliva motif [11]. Previous studies in rice, cotton and Arabidopsis have revealed that a type III secretion system of bacterial pathogens produced the transcription-activator-like (TAL) effector to target specific host *SWEET* genes and promote virulence [12,13,14,15,16]. Furthermore, the glucose transporter VvSWEET4 in grapevine was upregulated upon necrotroph fungal *Botrytis cinerea* infection, thus contributing to its virulence [17]. Therefore, SWEETs are likely hijacked by pathogens to benefit their pathogenicity during progression on host tissues.

A previous RNA-seq study showed that numerous maize genes were differently regulated upon *U. maydis* inoculation, and functional categories of DEGs were mostly enriched in defense and metabolic pathways [4]. Intriguingly, *U. maydis* infection could regulate a series of organ-specific genes in maize [2,18]. For example, *U. maydis* effector protein See1 induced maize cell cycle gene expression in leaves, leading to leaf cell division and eventually tumor formation [19]. Furthermore, maize-line-specific genes were also found to be involved in *U. maydis* and maize interaction [20]. Taken together, these findings indicate that the regulation of transcription reprogramming of host genes is critical for the biotrophic infection of *U. maydis*.

Plant cis-regulatory elements (CREs) are the key components of transcription reprogramming. CREs are usually located in accessible chromatin regions (ACRs) to regulate gene transcription via interaction with transcription factors in a tissue-specific manner [21]. Abundant CREs have been detected through accessible chromatin sequencing assays including MNase hypersensitive, DNase I hypersensitive, and transposase-accessible assays in the maize genome. Certain types of CREs are proven to contribute to tissue specific gene-expression [22,23,24,25]. Furthermore, ACRs are positively associated with gene expression levels, conserved noncoding sequences, transcription start sites (TSSs) and known transcription factor binding sites [22].

It is widely known that pathogen effectors could bind to host gene sequence and function as transcriptional activators to promote virulence [26]. For example, multiple effectors of the rice blast fungal pathogen *Magnaporthe oryzae* were secreted and translocated into the nuclei of host cells, and could subsequently reprogram the transcription of immunity-associated genes in host cells through binding to the specific motif in rice [27]. Similarly, biotrophic pathogen *U. maydis* infection in maize induced host transcription and metabolism reprogramming [5,28]. Despite these research advances, the mechanisms underlying the regulation of transcription reprogramming of maize genes by certain effectors of *U. maydis* to facilitate corn smut disease remain largely unexplored. In this study, we deployed multiple transcriptome datasets and a maize genetic population to identify specific CREs which might be involved in the transcription reprogramming of host genes during infection by *U. maydis*. Through a cis-element enrichment assay, we were able to identify a conserved cis-element RY-repeat in the promoter of multiple host genes, which has barely been reported to be involved in host–microbe interactions. Moreover, screening the yeast library and knocking out approaches enabled us to find a candidate-secreted protein UMAG_06102 of *U. maydis*. UMAG_06102 could interact with the RY-repeat-containing region of host genes in the Y1H system and might regulate their transcription reprograming, thus impacting its virulence during infection of host tissues.

## 2. Results

### 2.1. RY-Repeat Is Enriched in Up-Regulated Genes upon U. maydis Infection

**Figure 1:**
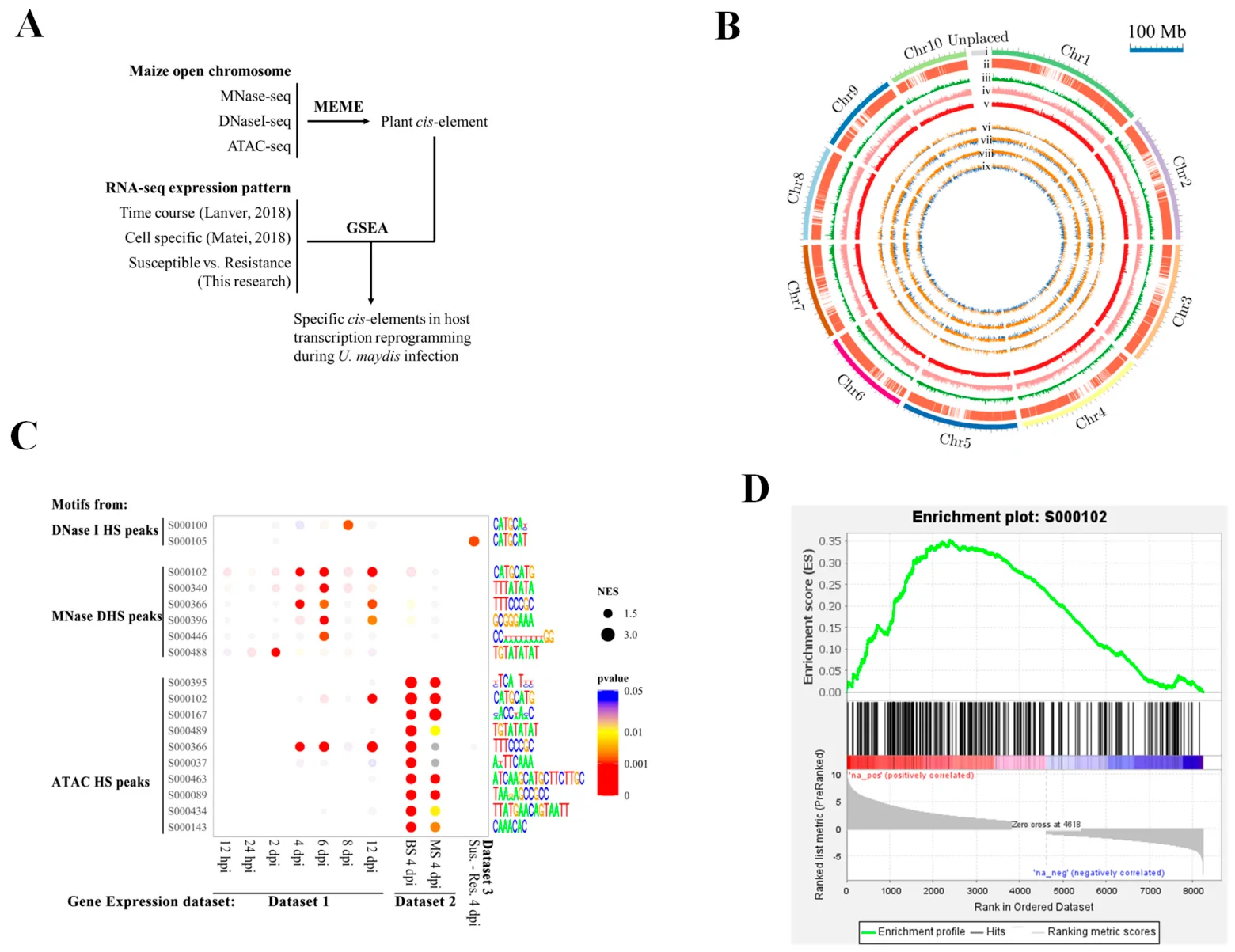
Cis-element enrichment analysis of maize genes. (**A**) Pipeline of plant cis-element annotation and GSEA. (**B**) Genome-wide visualization of open chromosome HS overlapped with gene TSS region in seedling leaves and aligned against other genomic features: (i) chromosome ideograms; (ii) gene density; (iii) DNaseI HSs overlapped with gene TSS region [29]); (iv) ATAC HSs overlapped with gene TSS region [24]; (v) MNase DHSs overlapped with gene TSS region [22]; (vi) gene expression fold change from RNA-seq of *U. maydis* infected and mock seedlings at 4 dpi (days post infection) [30]; (vii) gene expression fold change in hyperplasic tumor cell caused by *U. maydis* infection and mock bundle sheath cells at 4 dpi [31]; (viii) gene expression fold change in hypertrophic mesophyll tumor cell caused by *U. maydis* infection and mock mesophyll cells at 4 dpi [31]; and (ix) gene expression fold change in susceptible and resistant line at 4 dpi of *U. maydis* (this study). (**C**) Dot-plot of cis-element GSEA in *U. maydis*-induced maize genes across different datasets. The color of the dots represents the *p*-value of enrichment, and dot size represents the NES (normalized enrichment score); opaque and transparent dots represent data passing or not the FDR test, respectively. (**D**) Enrichment plot of MNase DHS annotated RY-repeat in 12 dpi RNA-seq dataset. The green line in the chart represents the enrichment score, black vertical lines indicate RY-repeat annotated genes hit in the RNA-seq dataset, and the histogram at the bottom of the chart shows the fold change value of genes from the RNA-seq dataset.

To characterize the cis-element regulation landscape of maize during *U. maydis* infection, we performed a meta-analysis using a series of ACR data to identify cis-elements using MEME approaches, which were then enriched by integrating a set of RNA-seq expression datasets post infection of *U. maydis* (Figure 1A). It clearly showed that the ACRs, including DNase I HSs, MNase DHSs and ATAC HSs, overlapped well with gene TSS evenly across each chromosome within entire genome (Figure 1B). GSEA of RNA-seq datasets from maize seedling leaves showed that numerous cis-elements were enriched in different combinations of ACR data and RNA-seq datasets, among which multiple cis-elements, such as S000100, S000105 and S000102, containing bidirectional CATG sequences, were most abundant in all enrichment data from the three RNA-seq datasets (Figure 1C). For instance, analysis of the MNase DHSs-annotated RY-repeat in the 12 dpi RNA-seq dataset showed that genes with the RY-repeat in their proximal promoter regions were enriched in the most up-regulated DEGs upon *U. maydis* infection (Figure 1D), but not enriched in up-regulated DEGs upon *Fusarium graminearum* infection (Figure S1A,B). Moreover, RY-repeat genes were mainly induced at the later stage of *U. maydis* infection, suggesting that RY-repeat might be associated with the pathogenicity of *U. maydis*.

### 2.2. ZmSWEET11 Is One of the Most Induced Genes upon U. maydis Infection

**Figure 2:**
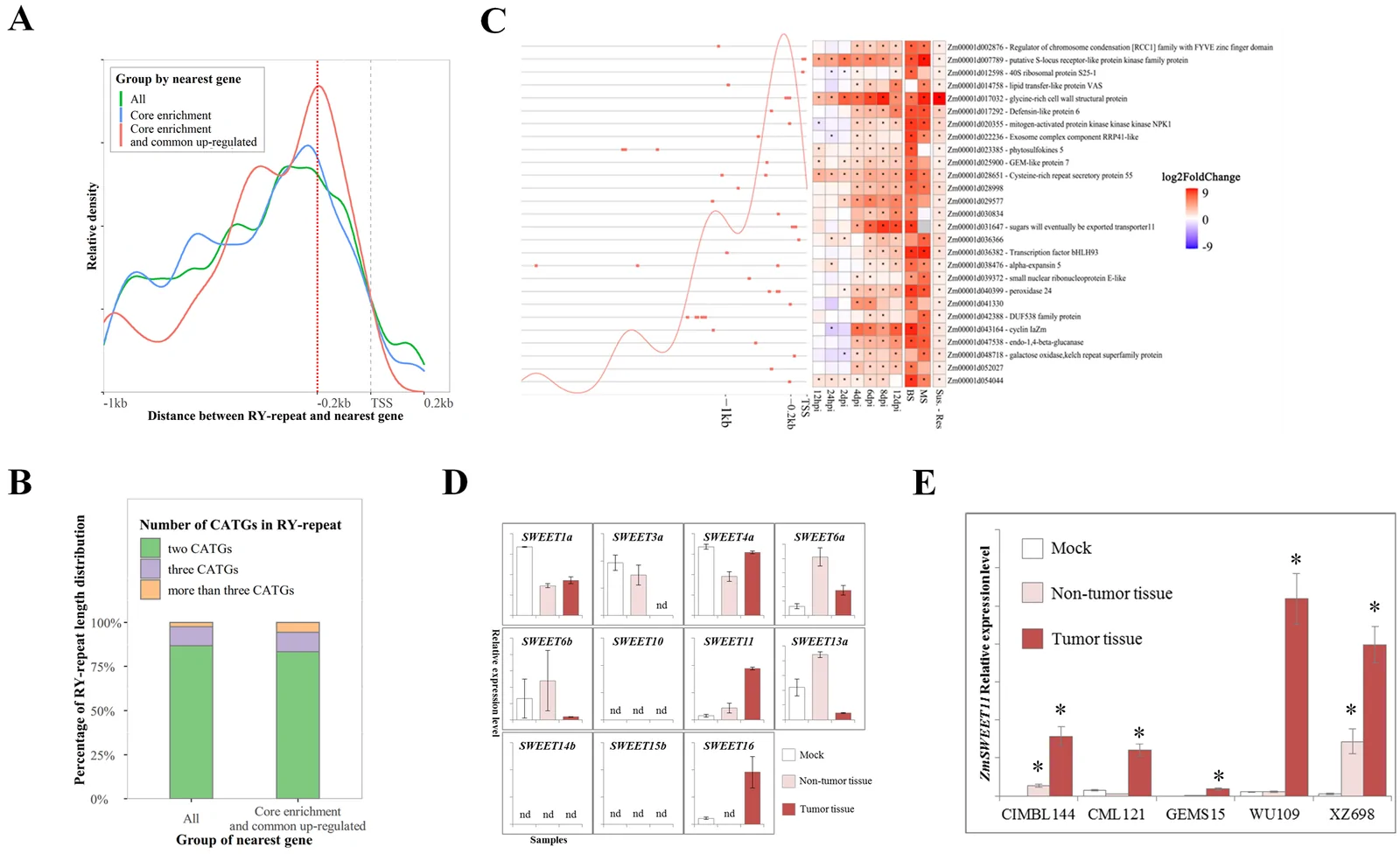
RY-repeat core enrichment gene analysis. (**A**) Relative density chart of the distribution of RY-repeat in RY-repeat annotated genes, RY-repeat core enrichment genes, and commonly up-regulated RY-repeat core enrichment genes, respectively, across three RNA-seq datasets. The red dashed line on horizontal axis indicates the peak of RY-repeat distribution distances from the TSS, and the TSS was marked by a gray dashed line on horizontal axis. (**B**) Histogram of the number of CATG function units in RY-repeat annotated genes, RY-repeat core enrichment genes, and commonly up-regulated RY-repeat core enrichment genes, respectively, across three RNA-seq datasets. (**C**) Left panel: Distribution of RY-repeat in promoter region of 27 commonly up-regulated RY-repeat core enrichment genes. Each horizontal line represents the upstream 0 to 3 kb region of TSS; the red boxes with different widths on the horizontal line indicate the RY-repeat with different CATG function units, and the red curve indicates the relative density chart in Figure 2A. Right panel: Gene expression foldchange heatmap of 27 commonly up-regulated RY-repeat core enrichment genes from three RNA-seq datasets. (**D**) Histogram of relative expression levels of maize *SWEET* gene family in mock, non-tumor leaf tissue, and tumor leaf tissue upon *U. maydis* infection at 8 dpi. The statistical significance was performed with Student’s *t*-test, and * *p* < 0.05 indicates significant difference compared with mock. nd, not detected. (**E**) Histogram of relative expression levels of *ZmSWEET11* in mock, non-tumor leaf tissue, and tumor leaf tissue upon *U. maydis* infection at 8 dpi in five pedigrees. The statistical significance was performed with Student’s *t*-test, and * *p* < 0.05 indicates significant difference compared with mock.

To better understand whether the RY-repeat genes are induced by *U. maydis* infection, 377 core enrichment genes were defined by GSEA as main contributors to the enrichment score, and 27 of them were significantly up-regulated in all RNA-seq datasets (Supplementary Table S1). Moreover, the RY-repeats of the core genes enriched and up-regulated in three RNA-seq datasets were mainly located at around 200 bp upstream of the TSS (Figure 2A). The comparison of the number of CATG motifs in RY-repeat showed that the core enrichment genes that were commonly up-regulated contained relatively higher numbers of CATG function units, e.g., more than three CATGs (Figure 2B).

Furthermore, analysis of the RNA-seq expression pattern of core enrichment genes showed that the 27 genes mentioned above displayed differential expression patterns in response to the infection with *U. maydis*, the majority of which were induced starting at 4 dpi. Intriguingly, among those genes, *ZmSWEET11* (*Zm000001d031647*) containing the RY-repeat with 4 CATG tandem repeats at 152 bp upstream of TSS was constantly significantly induced through 4 dpi to 12 dpi, and induced more than 200-fold compared with mock after 6 dpi (Figure 2C). In the RNA-seq datasets of BS (Bound sheath) vs. MS (Mesophyll-bound sheath), *ZmSWEET11* was only induced in BS cells, but not MS cells. Moreover, another gene, *glycine-rich cell wall structural protein* (*Zm00001d017032*), was also strongly induced in all datasets.

To address whether *ZmSWEET11* was specifically activated by infection with *U. maydis*, we first characterized the maize *SWEET* gene family and performed a phylogenetic tree analysis with that from *Arabidopsis thaliana* and rice (*Oryza sativa*). In total, 24 *ZmSWEETs* were identified and classified into four clades (Figure S2). We then conducted the expression analysis of the *SWEET* gene family in maize upon infection. Notably, *ZmSWEET11* and *ZmSWEET16*, but not other members of the maize *SWEET* gene family, were up-regulated in the samples with tumors at 8 dpi, yet only *ZmSWEET11* was significantly induced (Figure 2D). In line with this, *ZmSWEET11* was significantly induced in the tumor tissue of multiple lines (Figure 2E).

### 2.3. Y1H Library Screening Identified Proteins Interacting with ZmSWEET11 Promoter

To understand how *ZmSWEET11* is induced during *U. maydis* infection, we constructed the yeast one hybrid (Y1H) screening library with the total RNA of infected maize leaves and *U. maydis* hyphae, and used the promoter regions 42 bp to 852 bp and 42 bp to 402 bp upstream of *ZmSWEET11* TSS as bait sequences to screen the prey proteins. From more than 300 screened positive clones, three protein-encoded genes were selected based on their high frequency and positive complement verification. Among these three DNA binding proteins, two maize genes—*Germin-like Protein1* (*ZmGP1*, *Zm00001d037513*) and *Cellulose Containing Protein* (*ZmCCP*, *Zm00001d010039)*—and one hypothetical protein—(*UMAG_06102*) with glucanase domain from *U. maydis*—were identified, which prompted us to wonder how *ZmSWEET11* is regulated by this fungal protein.

**Figure 3:**
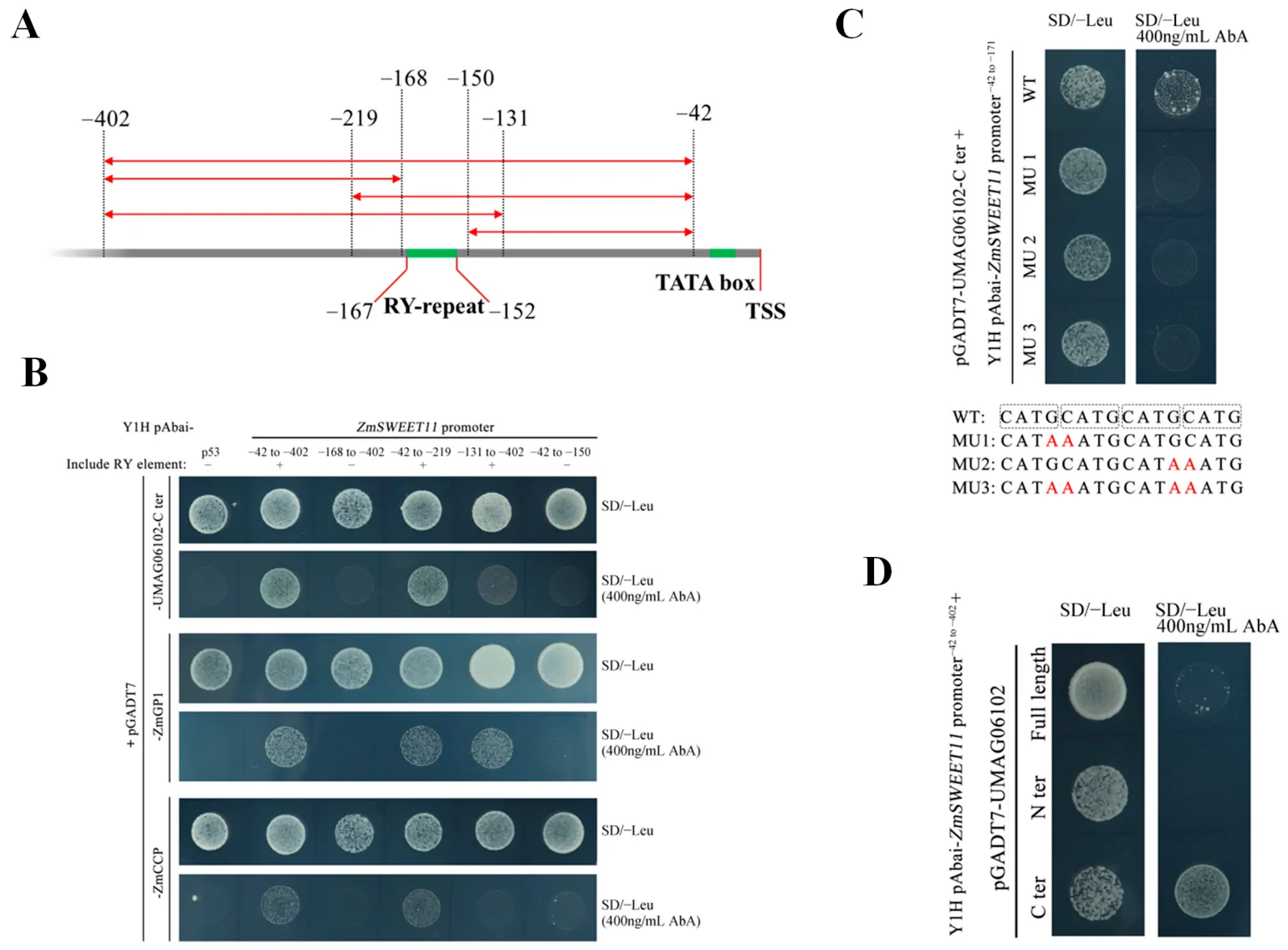
Yeast-one-hybrid assay for the interaction between *ZmSWEET11* promoter sequence and candidate proteins. (**A**) Structure of RY-repeat-containing fragments of *ZmSWEET11* promoter sequence tested in Y1H assay. (**B**) Y1H assay for the interaction between different *ZmSWEET11* promoter sequences with or w/o RY-repeat and UMAG_06102 C terminal, *ZmGP1* and *ZmCCP* proteins. The distinguished clones appeared on a plate with Aureobasidin A (AbA), indicating the interaction between RY-repeat and candidate protein. (**C**) Y1H interaction between UMAG_06102 C terminal and *ZmSWEET11* promoter sequence carrying mutation on RY-repeat sequence. Dotted boxes indicate the element CTAG repeat sequences. (**D**) Y1H interaction between *ZmSWEET11* promoter sequence and different fragments of UMAG_06102 protein.

In order to identify the binding sequences of the *ZmSWEET11* promoter by prey protein UMAG_06102, the *ZmSWEET11* promoter sequence was split into four different fragments (Figure 3A), where the first fragment, ranging from 168 bp to 402 bp, did not include the RY-repeat (152 bp to 167 bp), and the second fragment, ranging from 42 bp to 219 bp, covered the whole RY-repeat, while in the third fragment (131 bp to 402 bp), the RY-repeat was added to the first fragment, and in the fourth fragment (42 bp to 150 bp), the RY-repeat was removed from the second fragment. UMAG_06102 showed an interaction with the fragment containing the RY-repeat. However, the interaction between UMAG_06102 and the promoter was abolished when the RY-repeat was absent (Figure 3B).

To confirm the interaction of UMAG_06102 and the promoter of *ZmSWEET11*, we constructed several mutants of RY-repeat in the *ZmSWEET11* promoter, among which both the “G” in the first CTAG and “C” in the second CATG were changed to “A” in MU1; both the “G” in the third CTAG and the “C” in the fourth CATG were changed to “A” in MU2; and MU3 contained all mutations in both MU1 and MU2. The Y1H assay clearly showed that while mutations do not alter basal reporter activity independently of UMAG_06102, all mutants of the RY-repeat were deficient in the interaction of UMAG_06102 and *ZmSWEET11* promoter (Figure 3C). Moreover, the C-terminus but not N-terminus seemed to be required for this interaction (Figure 3D). Together, these data indicate that the RY-repeat was required for the Y1H interaction between the *ZmSWEET11* promoter and UMAG_06102.

### 2.4. Deletion of UMAG_06102 Is Associated with the Reduced Virulence of U. maydis and the Reduced Expression of RY-Repeat-Containing Genes

**Figure 4:**
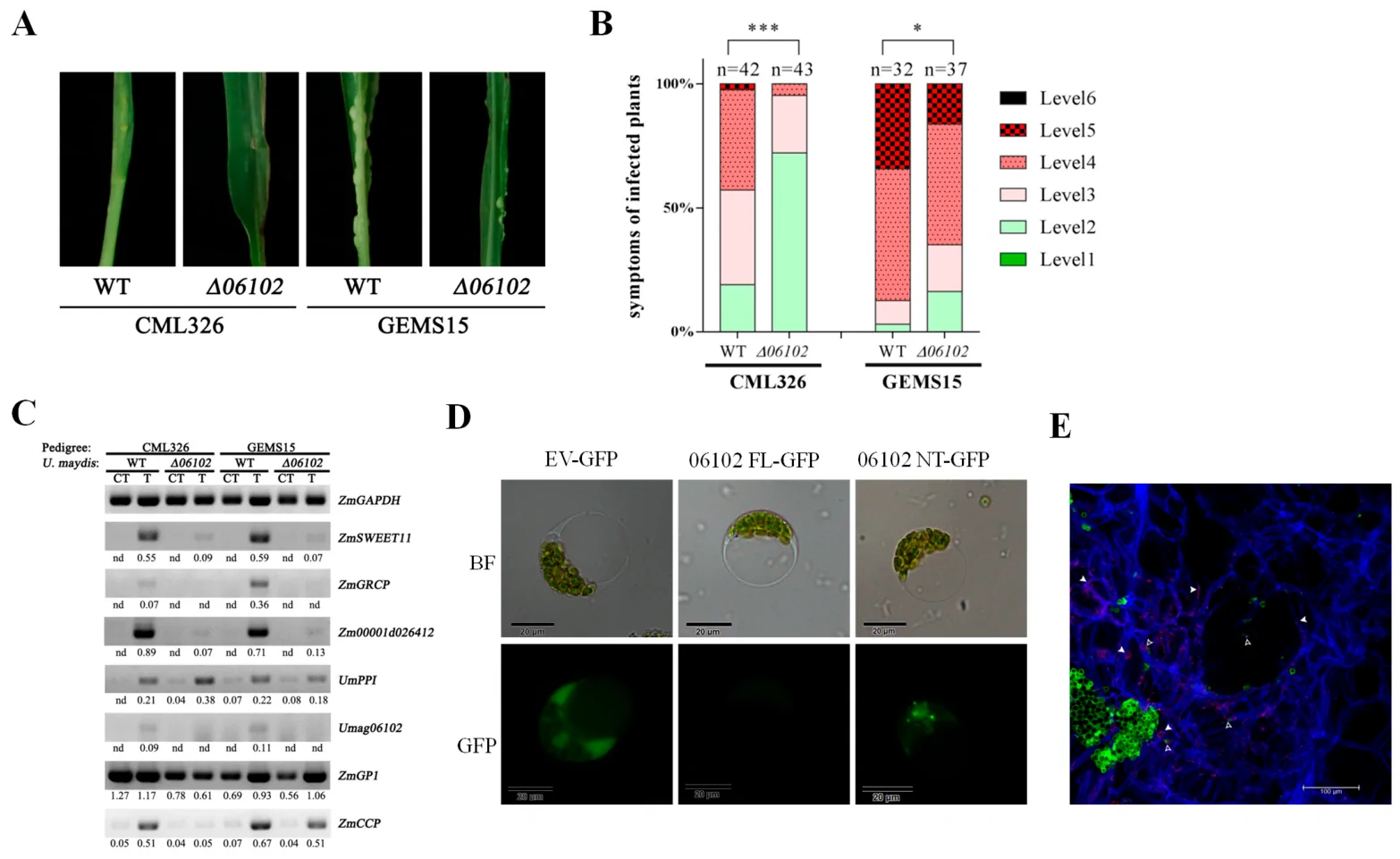
Deletion of UMAG_06102 is associated with reduced *U. maydis* virulence and reduced expression of RY-repeat-containing genes. (**A**) Disease symptoms of resistant maize line CML326 and susceptible line GEMS15 upon inoculation with *U. maydis* WT or *Δ06102* strain for 8 days. (**B**) Disease symptom index quantification in both lines upon inoculation with *U. maydis* WT or *Δ06102* strain for 8 days. The assay was performed three times independently, and disease symptom indexes were analyzed with a Kruskal–Wallis test between WT and *Δ06102*. * indicates *p* < 0.05, and *** indicates *p* < 0.001. (**C**) Semi-quantitative PCR analysis of genes in non-tumor (CT) and tumor (T) samples. The relative band density normalized by *ZmGAPDH* was quantified and labeled below the band. nd, not detected. (**D**) Subcellular localization of UMAG_06102 full length (FL) and N-terminal (NT, 1-283 amino acid) in maize protoplast. BF, bright field; EV, empty vector. Scale bar on the images = 20 μm. (**E**) Subcellular localization of *U. maydis* UMAG_06102::mCherry-HA in maize seedling leaf tissue at 12 dpi upon infection. The assay was conducted using a confocal microscope. The infection site of *U. maydis* was shown by an open arrowhead. The blue channel represented maize cell wall autofluorescence, the green channel *U. maydis* spores and hyphae stain with WGA-AF488, and the red channel mCherry signal tagged on UMAG_06102 (arrowheads). Scale bar on the image = 100 μm.

Given that UMAG_06102 showed a Y1H-detected interaction with the RY-repeat-containing region of the *ZmSWEET11* promoter, we evaluated the role of UMAG_06102 in transcription reprogramming of the RY-repeat-containing genes in maize and its impact on the regulation of *U. maydis* virulence. First, we screened out a pair of maize lines from a maize population. The line CML326 showed obvious resistance, while line GEMS15 was more susceptible to *U. maydis* (Figure S3A), with the former having significantly reduced disease levels 4 and 5, but increased levels 1~3. We then constructed the *UMAG_06102* knockout strain *Δ06102* of *U. maydis* and conducted the infection using both wild type (WT) and *Δ06102* mutant on these two lines. Compared to *U. maydis* WT strain, the *Δ06102* strain showed reduced virulence in both lines (Figure 4A). For instance, the resistant line CML326 displayed symptoms mostly ranging from level 2 (19.05%) to level 4 (40.48%) upon infection with WT strain, whereas the *Δ06102* strain brought the symptoms mainly down to level 2 (72.09%) and level 3 (23.26%). Similarly, the *U. maydis* WT strain caused disease symptoms mainly at level 4 (53.13%) and level 5 (34.38%), which were reduced to level 2 (16.22%), level 3 (18.92%), level 4 (48.65%) and level 5 (16.22%) when infected by the *Δ06102* strain (Figure 4B).

To examine whether the expression level of RY-repeat-containing genes was impacted by UMAG_06102, we collected seedlings with disease symptom at level 3 and level 4, and compared gene expression levels in non-tumor tissue and tumor tissue in the same leaf collected from both lines, upon infection with WT and *Δ06102* strain (Figure S3A). To verify the expression specificity of the RY-repeat-containing gene by UMAG_06102, two additional RY-repeat-containing genes, *ZmGRCP* (*Glycine-Rich Cell wall structural Protein*, *Zm00001d017032*) and *Zm00001d026412* (uncharacterized protein), were tested. Furthermore, *ZmGP1* and *ZmCCP* identified by Y1H were also included in the test. The relative expression level of *ZmSWEET11* examined by semiquantitative RT-PCR displayed a higher trend in tumor tissue but not in comparable non-tumor tissue. However, its expression appeared lower in the tissues inoculated with the *U. maydis Δ06102* strain than in WT-infected tissues (Figure 4C and Figure S3B). Similarly, the expression levels of the other two RY-repeat-containing genes, *ZmGRCP* and *Zm00001d026412*, also appeared lower in *Δ06102* strain-infected tissues, both in the resistant and susceptible lines, compared to that infected with WT strains (Figure S3B). Moreover, in the *Δ06102* strain, the *UMAG_06102* coding sequence was replaced by the deletion cassette; therefore, *UMAG_06102* transcripts were not detectable in *Δ06102*-infected samples (Figure 4C and Figure S3B). *UMAG_06102* shows the most correlation with the tested RY-repeat core enrichment genes. However, *ZmGP1* was expressed across all samples, and did not show obvious reduction in *Δ06102*-infected tissues (Figure 4C). The housekeeping gene *ZmGAPDH* of maize showed similar expression levels in both resistance and susceptible plants, respectively, upon infection with either WT or *Δ06102* strains. However, RT-qPCR with independent biological replicates, efficiency-validated primers, and statistical evaluation is required to confirm these expression changes.

To understand whether UMAG_06102 functions as a transcription protein, we investigated the subcellular localization of UMAG_06102 using maize protoplast cells. It was clearly shown that the N-terminal fragment of UMAG_06102 accumulated in the nucleus, whereas full-length localization was less conclusive (Figure 4D). To track whether UMAG_06102 was secreted into the cells of host tissues, we constructed the *U. maydis UMAG_06102*::*mCherry-HA* strain, in which *mCherry-HA* was fused to the C terminus of *UMAG_06102*. The fungal growth of the *UMAG_06102*::*mCherry-HA* strain was subsequently visualized in the inoculated leaf tissue using confocal microscopy at 12 dpi. WGA-AF488 staining signals showed that *U. maydis* fungal hyphae and teliospore parasites were present in leaf tissue, and the UMAG_06102::mCherry-HA signal was detected in the vicinity of infection sites of *U. maydis* (Figure 4E). Moreover, the *UMAG_06102::mCherry-HA* strain caused similar disease levels to the WT strain on B73 leaves, both resulting in significantly higher disease incidence compared to that caused by mutant *Δ06102* (Figure S4A). Since the *UMAG_06102::mCherry-HA* strain is a tagged WT variant, a complementary strain is required to affirm the virulence of *UMAG_06102*.

### 2.5. Association of Natural Variation in the RY-Repeat in Maize Genes with Susceptibility to U. maydis

**Figure 5:**
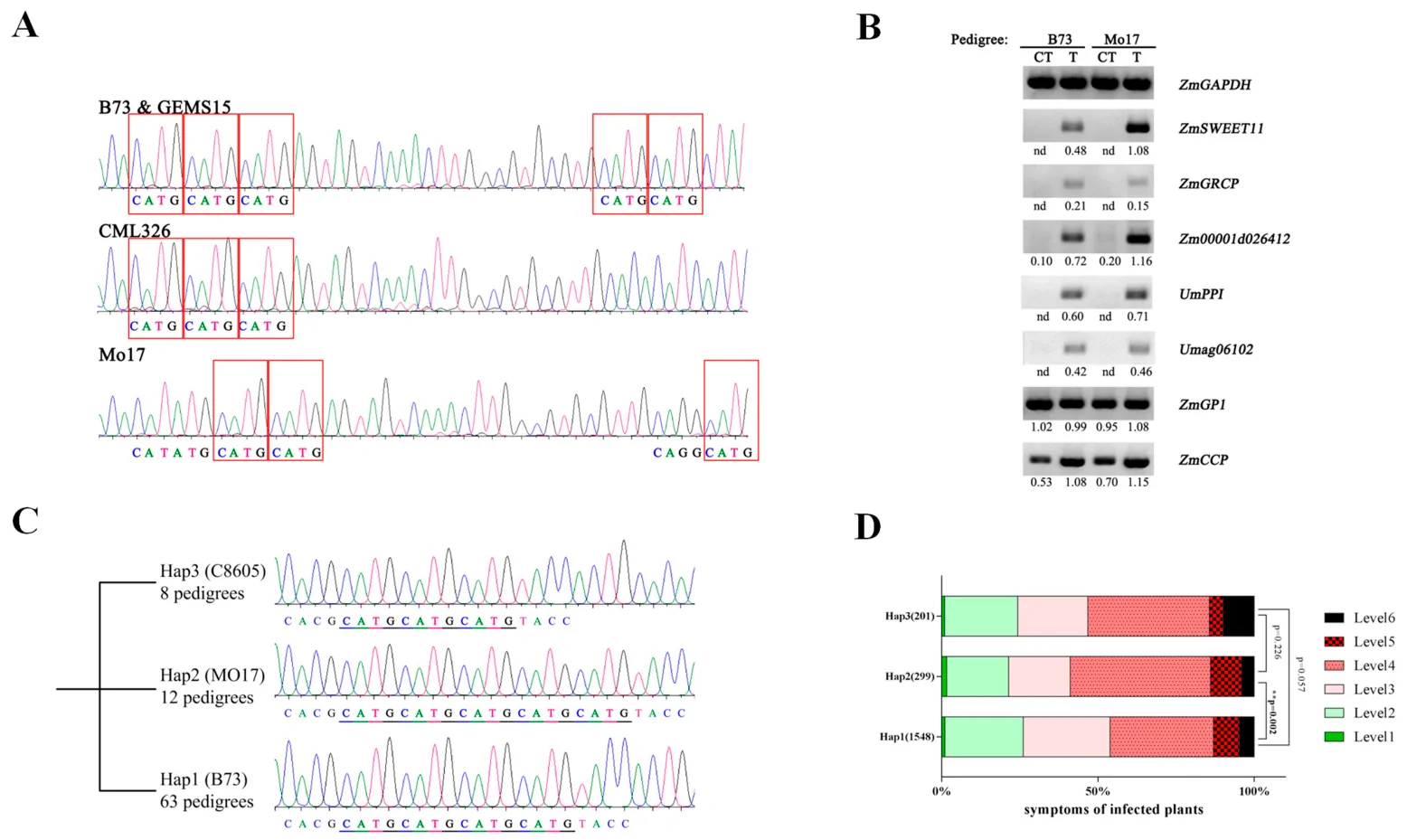
Correlation of natural variation in maize RY-repeat-containing genes with disease symptoms of *U. maydis*. (**A**) Natural variation in RY-repeat in *ZmGRCP* promoter across B73, GEMS15, CML326 and Mo17; red boxes indicate CATG function unit. (**B**) Semi-quantitative RT-PCR analysis of genes in B73 and Mo17 at 8 dpi of *U. maydis*. The relative band density normalized to *ZmGAPDH* was quantified and labeled below the band, and is shown for qualitative comparison only; no statistical conclusion is drawn. nd, not detected. (**C**) Haplotypes of RY-repeat in *ZmSWEET11* promoter across 83 pedigrees. The colored lines under the sequence indicate RY-repeats. (**D**) Disease symptom index distribution of three pooled haplotypes of maize lines at 8 dpi upon infection with *U. maydis*. Disease symptom index distribution results were analyzed with a Kruskal–Wallis test between haplotypes. ** indicates *p* < 0.01.

To understand whether the RY-repeat of maize genes is genetically associated with the susceptibility to *U. maydis*, we deployed a population consisting of 83 inbred maize lines, and identified natural variations in the above-mentioned RY-repeat-containing genes. Natural variations were found in the promoter of *ZmGRCP*, among which B73 and GEMS15 contain two RY-repeats, located at 212 bp to 219 bp and 243 bp to 254 bp upstream of TSS, respectively. Interestingly, line CML326 lost the RY-repeat at 212 bp to 219 bp, and line Mo17 carries two mutations, including two bases: AT insert at 253 bp and A to C mutation at 217 bp (Figure 5A). Semiquantitative RT-PCR analysis showed that the expression level of *ZmGRCP* was comparatively lower in the tumor tissue of Mo17, compared to B73, yet the expression levels of *ZmSWEET11* and *Zm00001d026412* were enhanced in Mo17 compared to B73 (Figure 5B). In line with this, the expression level of *ZmGRCP* in GEMS15, which carries complete RY-repeats, was comparatively higher than that in CML326, which was deficient in one RY-repeat in the *ZmGRCP* promoter (Figure 4C and Figure 5A,B).

Moreover, we sequenced the promoter region of *ZmSWEET11* among 83 inbred lines, and found that the complete RY-repeat was conserved in the *ZmSWEET11* promoter in all 83 lines, whereas the length of the RY-repeat was largely variable across different lines. Three haplotypes were found in the RY-repeat of the *ZmSWEET11* promoter, with a different number of CATG tandem repeats. Haplotype 1 (Hap1), containing four CATG tandem repeats, was found in 63 pedigrees including B73, CML326 and GEMS15, while 12 pedigrees including Mo17 containing five CATG tandem repeats were grouped into haplotype 2 (Hap2). The remaining eight pedigrees belonged to haplotype 3 (Hap3), containing three CATG tandem repeats (Figure 5C and Figure S5A).

To explore whether the disease severity of *U. maydis* differed among RY-repeat haplotype groups, we performed *U. maydis* assays on 83 pedigrees (Figure S5B), and the disease symptoms of pedigrees were pooled and compared according to three different haplotypes of the *ZmSWEET11* promoter. A Kruskal–Wallis test showed that the disease symptom distribution of Hap2 (five CATG tandem repeats) was significantly different from that of Hap1 (four CATG tandem repeats), with an increased percentage of plants showing heavy tumor (disease severity index level 5) observed in the Hap2 group. However, the percentage of plants with heavy tumor was decreased in Hap3 (three CATG tandem repeats) (Figure 5D).

## 3. Discussion

Corn smut caused by *U. maydi*s results in a significant yield loss of maize, and examining the interactions involved is a well-established model to understand the infection features of biotrophic pathogens. Upon infection on maize tissues, *U. maydis* mature teliospores are usually released into the surrounding soil, whereas *U. maydis* specifically infects aerial organs of maize. Accumulating evidence through transcriptome analysis have suggested that the transcription reprogramming of maize tissue-specific genes is required for successful colonization by *U. maydis* [2,18,20,32]. However, it remains largely unknown whether conserved plant cis-elements function in transcription reprogramming upon *U. maydis* infection, and whether *U. maydis* effectors could interfere in this transcription reprogramming process.

### 3.1. Uniqueness of RY-Repeat Enrichment in Response to U. maydis Infection

A previous study showed that gene expression but not repression could be mediated by the activation of cis-elements [33]. We scanned 469 identified plant cis-elements in three datasets of chromatin-accessible regions of maize seedling leaves, and filtered genes containing cis-elements near their TSSs. Through GSEA of three RNA-seq datasets, we identified numerous genes significantly enriched in the up-regulated DEGs, which is consistent with the organ-specific transcriptome data of *U. maydis*-inoculated tissue [2]. In that study, the organ-specific fungal DEGs were positively correlated with those of organ-specific DEGs, showing up-regulation instead of down-regulation in maize.

Intriguingly, the majority of up-regulated DEGs contain several specific cis-elements, among which the RY-repeat was significantly enriched in all three datasets of ACRs, and all three RNA-seq datasets. It has been reported that the RY-repeat was conserved in many seed-specific promoters, and could be activated by interaction with the B3 domain family protein, and RY-repeat-containing genes were specifically induced during embryo maturation, while being repressed in non-seed tissue and at the seedling growth stage [34,35,36]. However, the role of the RY-repeat involved in plant–pathogen interactions has not been fully investigated. A previous study showed that during early stages of *Ustilaginoidea virens* infection in rice, the RY-repeat motif was significantly enriched in the 5′-regulatory regions of a series of *pathogenesis-related* (*PR*) genes, which have been well known as marker genes associated with plant defense response [37]. In this study, 377 core enrichment RY-repeat genes were induced upon *U. maydis* infection, 27 of which were significantly up-regulated in all three RNA-seq datasets (Supplementary Table S1). Despite that, as the FDR < 0.25 threshold is relatively permissive, RY-repeat enrichment should be considered an exploratory candidate rather than a statistically confirmed discovery.

The RY-repeat in these commonly up-regulated genes was mainly located around 200 bp upstream of the TSS, which is consistent with previous findings in maize: that genes with proximal DHSs within 200 bp upstream of TSSs showed relatively higher expression levels [23]. This is also similar to the cis-element distribution of the VP1/ABA-regulated genes [33]. Moreover, we found that although few genes contain more than two CATG function units in the promoter region, the commonly up-regulated genes contain more CATG function units, similar to the findings of a previous study where the reduced number of CATG function units resulted in lower RY-repeat promoter activity [35].

Interestingly, the cis-element enrichment analysis of the RNA-seq dataset using maize seedlings inoculated with *F. graminearum* at 48 hpi clearly showed that the RY-repeat was not enriched upon *F. graminearum* infection. Furthermore, the RY-repeat was enriched at the biotrophic stage of *U. maydis* infection, in which many effectors were secreted into the host [38]. Therefore, the RY-repeat could be enriched in maize seedlings upon *U. maydis* infection but not in the *F. graminearum* dataset analyzed here, which was unrecognized before during the interaction between maize and pathogens. Nevertheless, a single comparison with *F. graminearum* is insufficient to establish the specificity of RY-repeat activation by *U. maydis*. Additional datasets from other maize fungal pathogens would provide stronger support for this conclusion.

Besides the RY-repeat, several other plant cis-elements were also enriched in up-regulated genes upon *U. maydis* infection in maize seedlings. For instance, the E2Fa-element containing TTTCCCGC motif was specifically and significantly enriched at 8 dpi. It is known that the E2Fa-element is related to the regulation of genes involved in cell cycle and cell division in tobacco, Arabidopsis and maize [39,40,41], suggesting that E2Fa-element activation might be correlated with tumor formation in the host tissue. Another cis-element, phyA-element containing TGTATATAT motif, was enriched at an early stage (2 dpi) of *U. maydis* infection. Moreover, phyA-element was reported to be associated with phytochrome A-regulated genes in Arabidopsis [42], indicating its possible role in the regulation of photosynthesis at an early stage of *U. maydis* infection. Therefore, it will be interesting to investigate how photosynthesis and cell cycle via regulation of cis-elements are correlated with the biological infection process of *U. maydis* on maize seedlings.

### 3.2. The Candidate Secreted Protein UMAG_06102 Is Associated with the Virulence of U. maydis on Maize Seedling

Y1H library screening showed that UMAG_06102 could interact with the promoter of *ZmSWEET11*, and the RY-repeat in the promoter sequence was associated with disease progression. UMAG_06102 carries a β-1,3-exoglucanase domain on the C terminal, and an *UMAG_06102::mCherry-HA* signal was detected in the vicinity of infection sites, which suggests that it may function as a cell wall degradation enzyme (CWDE). However, this signal alone cannot distinguish apoplastic from intracellular localization. The presence of a β-1,3-exoglucanase domain does not sufficiently demonstrate secretion. UMAG_06102 lacks a canonical signal peptide, and its secretion and translocation require additional experimental validation. CWDEs are usually the proteins secreted by pathogens, and they interfere with the host tissues, as well as promoting the retrieval of nutrients. Although 73 predicted CWDEs have been found in *U. maydis*, and transcriptome analysis revealed the specific expression pattern of CWDEs that occurred at pathogenic stages [43], the roles of CWDEs in the virulence of *U. maydis* remains unclear. *U. maydis* glucanase proteins UMAG_05223 and UMAG_05495 were specifically induced during the infection of maize seedlings, but neither one showed virulence defection in the knockout strain, which might be due to duplicated members of CWDEs in the *U. maydis* genome [18]. The *Δ06102* strain in this study showed reduced virulence in maize seedlings across pedigrees, suggesting that besides being CWDE, UMAG_06102 might also function through influencing host gene expression during *U. maydis* infection, as the UMAG_06102::mCherry signal was not only distributed along the host–cell wall, but also possibly inside the apoplasts. Dual-function effectors have previously been reported in *U. maydis*; for example, the *U. maydis* effector UmFly1 was a fungalysin functioning in both fungus and the maize host to promote virulence [43]. Although UMAG_06102 contains a putative β-1,3-exoglucanase domain, we cannot deny that the reduced virulence of *Δ06102* is partly due to altered fungal physiology, including factors such as growth, cell wall integrity, stress response, sporulation, mating or appressorium formation. These phenotypes were not assessed in this study and should be examined in future work.

Reduced Y1H interaction between full-length UMAG_06102 and the *ZmSWEET11* promoter indicated the secreted signal related to the N terminus of UMAG_06102; however, UMAG_06102 lacks a typical secreted signal peptide. Whether full-length UMAG_06102 enters the host nucleus, and whether proteolytic processing releases a C-terminal DNA-binding fragment, remains an open question. Until nuclear localization of the full-length protein is demonstrated, direct regulation of the *ZmSWEET11* promoter in the nucleus should be considered a hypothesis. Moreover, the mCherry signal alone cannot distinguish apoplastic from intracellular localization, nor can it demonstrate secretion, and the absence of a canonical signal peptide and the limited resolution of mCherry imaging preclude a definitive conclusion on secretion and translocation. Additional controls and more rigorous translocation assays are required to validate the nuclear localization of UMAG_06102 in maize cells.

Numerous studies on the interaction between transcription factors from pathogens and the DNA binding motif in host genes have revealed the mechanism of host transcription reprogramming. For example, TAL-effectors from *Xanthomonas* acted as transcriptional activators and directly bound to the plant host gene promoter, thus promoting virulence [26]. In addition, two secreted proteins from the fungal pathogen *Magnaporthe oryzae* were found to bind with a specific motif in the host and regulate host gene transcription [27]. Intriguingly, *U. maydis* effector Tip6 (Topless (TPL) interacting protein 6) could recruit maize coreceptor RELK2 through interaction with its Ethylene-responsive element binding factor-associated Amphiphilic Repression (EAR) motifs to disrupt the host transcriptional regulation network, thereby promoting *U. maydis* pathogenicity [44]. In this study, a comparison between the induction of host RY-repeat-containing genes upon infection with WT and *Δ06102* strain showed that, although similar expression levels of the *U. maydis* housekeeping gene were detected, the induction of the host RY-repeat-containing genes was suppressed in the samples infected with *Δ06102* strain, indicating potential Y1H-detected interaction with the RY-repeat in maize by the candidate secreted protein UMAG_06102. However, a polar effect of the deletion cassette on neighboring genes cannot be excluded, as the knockout was confirmed by PCR only. Full locus characterization and complementation are needed. Moreover, the direct binding of UMAG_06102 to the RY-repeat in planta remains to be validated by independent physical assays such as EMSA, DAP-seq or ChIP, as well as by promoter-reporter assays with intact and mutated RY-repeats in the future.

Surprisingly, the induction of the host RY-repeat was not fully abolished upon infection with the *Δ06102* strain, indicating that other factors might also be involved in the transcription reprogramming of RY-repeat-containing genes. For example, the expression levels of host *ZmGP1* and *ZmCCP*, two genes that also interacted with the *ZmSWEET11* promoter, were also altered upon *U. maydis* infection. The observation that the C-terminus mediates DNA binding in Y1H while the N-terminus shows nuclear accumulation raises the possibility of proteolytic processing or independent domain functions. Whether full-length *UMAG_06102* is cleaved to release a C-terminal DNA-binding fragment remains to be tested. Moreover, the distinct observations of C-terminal Y1H binding and N-terminal nuclear accumulation suggest a dynamic complex rather than simply the direct regulation of UMAG_06102 on the *ZmSWEET11* promoter. Furthermore, RNA-seq data indicated that *ZmSWEET11* was specifically induced in BS cells, which is distinct from other RY-repeat-containing genes; therefore, the reprogramming of RY-repeat-containing genes in maize host upon *U. maydis* infection could be exerted by multiple components, such as *U. maydis* effectors, host tissue type, and probably environmental conditions. UMAG_06102-related virulence of *U. maydis* in other host organs needs to be further investigated. Furthermore, due to the lack of a complemented strain, the reduced virulence of *Δ06102* cannot be unambiguously attributed to loss of *UMAG_06102*, and full genetic complementation of *Δ06102* by reintroducing *UMAG_06102* and demonstrating restoration of virulence and target gene expression is required to definitively attribute the phenotype to loss of *UMAG_06102*.

The source–sink balance is important not only for the growth and development of plants, but also for pathogen progression. Seeds are typical sink organs that rely on carbohydrate, nitrogen and other nutrient supplies from source organs [45,46]. During the infection of maize seedlings with *U. maydis*, the photosynthesis of infected leaves is repressed and cell proliferation is induced at a later stage, which indicates a reprogrammed source–sink balance. It is known that the sink strength of infected leaves and the efflux from other source leaves can be elevated in order to provide nutrients for the tumor formation of *U. maydis* [28,30]. Our data showed that phyA-element was related to photosynthesis, E2Fa-element was related to the cell cycle, and the seed-specific RY-repeat was also enriched in host up-regulated genes upon *U. maydis* infection, especially the RY-repeat-containing *ZmSWEET11*, which is likely to be involved in sugar transport during *U. maydis* infection. Nevertheless, we could not exclude the possibility that the reduced expression of *ZmSWEET11* and other RY-repeat-containing genes in *Δ06102*-infected tissues may be a secondary consequence of reduced disease severity and altered tumor formation. Quantitative fungal biomass measurements and promoter-reporter assays are needed to distinguish direct regulation from indirect effects.

### 3.3. ZmSWEET11 Might Contribute to the Carbohydrate Supply for the Pathogenicity of U. maydis

Previous work using single-cell RNA-seq revealed that *ZmSWEET13a*, *b*, and *c* were specifically expressed in abaxial BS cells in maize leaves, which was highly correlated with the sucrose transfer towards the phloem [47], indicating that SWEET proteins might play important roles in the acquisition of apoplasmic carbohydrates in maize leaf tissues. In this study, we found that the expression levels of three *ZmSWEET13s* were repressed upon *U. maydis* inoculation, while the *ZmSWEET11* in same subgroup was greatly induced (Figure 3D), and a cell-specific RNA-seq dataset showed that *ZmSWEET11* was specifically induced in BS cells but not MS cells. We hypothesized that, upon *U. maydis* infection, the suppression of *ZmSWEET13s* might inhibit the transfer of carbohydrates towards the pathogen, whereas the induction of *ZmSWEET11* in BS cells might contribute to the pathogenicity of the fungus through sucrose efflux from the host to pathogen [48]. Interestingly, we found that the tumors formed on maize leaves tended to be distributed on the abaxial side of the leaf tissue (Supplementary Figure S6B), suggesting the possibility that the specific expression of *ZmSWEET* and sucrose efflux was more abundant in abaxial BS cells. Moreover, the genetic study showed that greater numbers of CATG function units were correlated with increased tumor formation on leaf tissue, suggesting that *ZmSWEET11* could likely act as a disease-susceptibility factor in maize and *U. maydis* interactions. However, the association between *ZmSWEET11* promoter haplotypes and disease severity is based on a small and unbalanced panel and does not account for population structure or kinship. A mixed-model association analysis with principal components/kinship is required to confirm this relationship.

Accumulating evidence has clearly showed that host sugars and their transporter proteins play important roles in host–pathogen interactions, very likely being hijacked by pathogens as nutrients to promote pathogenicity [48,49,50,51]. Interestingly, SWEET protein was also reported to function as a nutrient supplier for arbuscular mycorrhizal (AM) symbiosis in *Medicago truncatula* [52]. Therefore, attention was drawn to the creation of new varieties via targeting these unique cis-elements of *SWEET* genes to enhance disease resistance to pathogens [53]. Indeed, in rice, a specific effector-binding element (EBE) in the promoter of *OsSWEET14* (also known as *Os11N3*), the well-defined rice bacterial blight susceptible gene, was targeted by TAL effectors, which prompted successful disruption through TALENs [54]. Two more recently published works simultaneously deployed CRISPR-Cas9 technology to disrupt the TALE-binding elements (EBEs) of two rice *SWEET* genes, *OsSWEET11* and *OsSWEET14*. Those two genes both functioned as susceptible factors to facilitate the disease development of *Xanthomonas oryzae pv. oryzae* (*Xoo*), and also as targets to generate novel lines with broad-spectrum resistance against multiple *Xoo* strains [55,56]. Therefore, the finding of the RY-repeat in *ZmSWEET11*, as well as in other genes, could open a door for engineering novel maize varieties with enhanced resistance to *U. maydis* by targeting this candidate susceptibility-associated gene via genome editing. Transient silencing or genome editing of *ZmSWEET11*, followed by disease severity assessment, is required to fully establish its role in *U. maydis* tumor formation in the future.

## 4. Materials and Methods

### 4.1. Plant Materials, Fungal Cultivation and Infection

Maize lines used in this study were provided by Prof. Jianbing Yan at Huazhong Agricultural University, and the seeds were sown and grown in potting soil (garden soil:vermiculite = 1:1). The seedlings were grown in a temperature-controlled greenhouse with a 14-/10 h light–dark photoperiod, with the light flux density at 300 μmol·m^−2^·s^−1^ and a temperature of 28/20 °C (day/night).

The *U. maydis* strain was isolated and provided by Prof. Canxin Duan at the Chinese Academy of Agricultural Sciences from infected plants from a field in Hebei province. The strains were isolated from field-collected fungal spores. Briefly, 0.05 g of spore powder was evenly spread on sterile filter paper moistened with sterile distilled water in a 90 mm Petri dish and incubated at 28 °C for 72 h to allow spore germination. The germinated spores were rinsed with 1 mL sterile distilled water, and the resulting suspension was filtered through a 40 μm cell strainer (BD Falcon). The filtrate was diluted 100-fold, and 200 μL was spread onto PDA plates supplemented with ampicillin (100 μg/mL) and kanamycin sulfate (50 μg/mL), followed by inverted incubation at 28 °C. After 2 days, single colonies appeared; individual colonies were picked, numbered, and inoculated into 3 mL YEPSL medium at 28 °C with shaking at 200 rpm. After 1 day, the cultures became turbid. Spore morphology was examined under a microscope, and the cultures were centrifuged at 3000 rpm for 15 min at room temperature. The supernatant was discarded, and the spore pellet was resuspended in 1 mL YEPSL medium containing 25% glycerol and stored at −80 °C.

For genotyping, isolated strains were cultured in 3 mL YEPSL medium at 28 °C with shaking at 200 rpm for 1 day. Cells were collected by centrifugation at 3000 rpm for 15 min at room temperature, and genomic DNA was extracted using a modified CTAB method. Briefly, the pellet was resuspended in 500 μL pre-warmed (65 °C) 2% CTAB extraction buffer and incubated at 65 °C for 1 h. An equal volume of chloroform (500 μL) was added, mixed thoroughly, and centrifuged at 12,000 rpm for 10 min at 4 °C. Approximately 400 μL of the upper aqueous phase was transferred to a clean 1.5 mL tube, mixed with 400 μL isopropanol, and centrifuged at 12,000 rpm for 1 min at 4 °C. The supernatant was discarded, and the DNA pellet was washed twice with 500 μL 70% ethanol, followed by centrifugation at 7500 rpm for 1 min at 4 °C. After air-drying to remove residual ethanol, the DNA was dissolved in 30 μL ddH_2_O and its concentration and A260/280 ratio were measured using a Nanodrop spectrophotometer.

Mating-type loci were genotyped by PCR using Phanta Max Super-Fidelity DNA Polymerase (Vazyme, P505) with locus-specific primers. Primers UMfa1-F/UMfa1-R specifically amplify a 549 bp fragment from strains carrying the *a1* allele (annealing at 58 °C, extension for 1 min), whereas primers UMfa2-F/UMfa2-R specifically amplify a 637 bp fragment from strains carrying the *a2* allele (annealing at 58 °C, extension for 1 min). Primers UMb1-F2/UMb1-R7 amplify an approximately 1296 bp fragment from the *b* locus across different genotypes (annealing at 58 °C, extension for 1 min 30 s). The *b* locus genotype was determined by sequencing and alignment of the amplified products.

Two compatible mating-type strains of *U. maydis* were used in this study, one carrying the *a1b3* genotype and the other carrying the *a2b2* genotype (Supplemental Figure S7). The strains were added to 3 mL of YEPSL medium (0.4% yeast extract [*w/v*], 0.4% bactopeptone [*w/v*], and 2% sucrose [*w/v*]), followed by culturing overnight at 28 °C with shaking at 200 rpm, respectively. After a microscopic examination of spore morphology, the culture was transferred into 30 mL of YEPSL medium and further cultivated at 28 °C with shaking at 200 rpm for 1–2 days until the OD600 of the culture reached 1.0. The culture was then centrifuged at 3000 rpm for 15 min at room temperature, and the supernatant was discarded. The spores of two mating-type samples were resuspended in 20 mL of sterile water, respectively, and the spore concentration was determined under a microscope. The spore suspensions of the two strains were adjusted to 10^7^ spores/mL with sterile water separately, then mixed in equal volumes and supplemented with Tween 20 to a final concentration of 0.01%. The final concentration of each strain in the mixed inoculum was 5 × 10^6^ spores/mL. The mixture was homogenized and the fungal spore suspension was immediately injected with a syringe into the stem of the 7-day-old maize seedling, and the disease symptoms of individual plants were scored at 8 days post infection (dpi), according to a previous description [57] (Figure S6).

### 4.2. Fungal Strain Construction

*U. maydis* strains in this study were generated from wild-type *U. maydis* as described above. To knock out *UMAG_06102*, the sequences of 1kb upstream of *UMAG_06102* TSS, *U. maydis* hsp70 promoter, hygromycin B1 resistance gene, and 1 kb downstream of *UMAG_06102* stop codon were fused. To construct the mCherry-HA tagged UMAG_06102 stain, the sequences of 1 kb 3′ end of *UMAG_06102*, *mCherry* and *HA*, *U. maydis hsp70* promoter, *hygromycin B1 resistance* gene, and 1kb downstream of *UMAG_06102* stop codon were fused. The fusion construct was transformed into *U. maydis* protoplast as described previously [58]. The positive clones were selected on a YEPS light (1% yeast extract [*w/v*], 0.4% bactopeptone [*w/v*], and 0.4% sucrose [*w/v*], 2% agar [*w/v*]) plate containing hygromycin B1 (400 μg/mL), and confirmed by PCR. PCR primers are listed in Supplementary Table S2.

### 4.3. RNA-Seq Analysis

Maize stems of resistant-line CML326 and susceptible-line GEMS15 were infected with *U. maydis* at 1 h before the light cycle as described above, and then collected at 4 dpi. The mock treatment using water without fungal spore suspension was used as the control for each line. The experiments were replicated three times, with each treatment containing six plants. All three biological samples were ground in liquid nitrogen, and the total RNA was extracted with TRIZOL (Invitrogen) by following the manufacturer’s instructions.

Total RNA was subjected to quality control prior to library construction using Illumina TruSeq RNA library prep kit v2. Subsequently, RNA sequencing was performed using Illumina HiSeq 2000 (Illumina Inc., Beijing, China) at Berry Company, Beijing. Hisat2 software version 2.1.0 and Stringtie software version 1.3.5 were applied to map clean read pairs and for assembly with the B73 v4.59 reference genome [59,60,61], respectively. Gene count data were processed with R package DESeq2 [61]. Finally, two independent DESeq2 contrasts were performed: (i) CML326-infected vs. CML326-mock and (ii) GEMS15-infected vs. GEMS15-mock. Each genotype was compared with its own mock control. DEGs (differentially expressed genes) were filtered with the parameters of *p.* adjust < 0.05 and absolute log2FoldChange > 1 for each contrast separately.

### 4.4. Open Chromatin Data Processing and GSEA

The proximal promoter of protein coding genes (PCGs) was determined by including 1000 bp upstream and 200 bp downstream from the TSS [25]. ACRs from three maize seedling leaf datasets overlapped with PCGs were filtered [22,24,25], and then processed to analyze the occurrence of plant cis-acting regulatory DNA elements (https://rapdb.dna.naro.go.jp/PLACE/?action=newplace, accessed on 25 October 2020) with the MEME suite FIMO program [62,63]. DEG datasets identified from three RNA-seq analyses [30,31] were processed using the Gene Set Enrichment Analysis (GSEA) program with cis-element grouped gene sets, and the FDR threshold was set at 0.25 for the significantly enriched cis-element, as suggested [64]. Specifically, two contrasts frm the RNA-seq experiment (CML326-infected vs. CML326-mock and GEMS15-infected vs. GEMS15-mock) in this study were used as separate ranked gene lists for GSEA. Furthermore, two external RNA-seq datasets were used for GSEA: (i) *U. maydis*-infected vs. mock maize seedlings at 4 dpi [30]; (ii) hyperplastic tumor cells vs. mock bundle sheath cells at 4 dpi [31]; and (iii) hypertrophic mesophyll tumor cells vs. mock mesophyll cells at 4 dpi [31]. Each dataset was analyzed separately, and no cross-dataset contrast was performed. In brief, for each RNA-seq dataset, genes were ranked by their correlation with the phenotype of interest (infected vs. mock or susceptible vs. resistant), using the log2FoldChange as the ranking metric. Specifically, two independent DESeq2 contrasts were performed: (i) CML326-infected vs. CML326-mock, and (ii) GEMS15-infected vs. GEMS15-mock. Each genotype was therefore compared with its own mock control. DEGs were then filtered with *p*. adjust < 0.05 and log2FoldChange > 1 for each contrast separately. For GSEA, the log2FoldChange values from the CML326-infected vs. CML326-mock contrast and from the GEMS15-infected vs. GEMS15-mock contrast were used as separate ranked gene lists. Only one probe per gene was retained to avoid overestimation of the enrichment statistic. Only gene sets with at least 15 members were retained, consistent with the recommendation to focus on robust signals. The enrichment score was computed with the weighting exponent *p* = 1; the 469 cis-elements were scanned against ACR-defined proximal promoters (−1000 bp to +200 bp around the TSS) using MEME/FIMO (E-value < 1 × 10^−4^), and motifs were required to be detected in at least two of the three ACR datasets. Significance was estimated from 1000 phenotype-label permutations, with NES computed by rescaling positive and negative scores separately, and FDR q-values were derived from the null distribution of NES (S, π) across all gene sets and permutations. Stability was verified by the consistent enrichment of the RY-repeat across all three ACR datasets and all three RNA-seq datasets.

### 4.5. Quantitative Real-Time PCR and Semi-Quantitative PCR

Total RNA samples were reverse-transcribed to cDNA using the HiScript II Q RT SuperMix kit (Vazyme, cat R223-01). The relative expression levels of selected genes were detected by reverse transcription quantitative real-time PCR (RT-qPCR) using the AceQ qPCR SYBR Green Master Mix (Vazyme, catalog No. Q111-02) and BioRad CFX96 Real-Time PCR Detection System. *ZmGAPDH* was used as a housekeeping gene for maize and *UmPPI* as a relative reference for *U. maydis*, respectively. The relative expression levels of target genes were calculated using the 2-ΔΔCT method. For semi-quantitative PCR, 10–50 ng cDNA was used as the template, and 300~600 bp fragments were amplified with Taq DNA polymerase (Vazyme, catalog No. P102-02) and specific primers. Gene expression levels were determined with ImageJ Gel Analyzer (v1.51), based on the band intensity on the agarose gel (Tsingke, catalog No. TSJ001). Gene-specific primers are listed in Supplementary Table S2.

### 4.6. Yeast-One-Hybrid Library Construction and Screening

To construct the yeast library, total RNA was extracted from maize leaf tissues with tumors at 8 dpi upon infection with *U. maydis* and *U. maydis* hyphae grown on maize seedling leaf tissues for 2 days, respectively. The sequences 42 bp to 852 bp and 42 bp to 402 bp upstream TSS of *ZmSWEET11* were cloned as bait sequences, and yeast-one-hybrid library screening was prepared and performed by following the manufacturer’s instructions according to the Matchmaker Gold Y1H Library Screening System (Clontech, catalog No. 630491). We pretested each bait construct in a series of concentrations of Aureobasidin A (AbA), at 50, 100, 200, 400 and 800 ng/mL, respectively, to determine the minimal concentration that fully suppressed autoactivation, and 400 ng/mL AbA was used in the following experiments.

To confirm the interaction of UMAG_06102 with RY-repeat, Y1H assays were performed using the Matchmaker Gold Yeast One-Hybrid System (Clontech/Takara) according to the manufacturer’s instructions. The pAbAi-bait constructs were linearized and integrated into the Y1HGold strain to generate bait-specific reporter strains. To check non-specific activation by the GAL4 activation domain, the empty pGADT7 vector was co-transformed with each pAbAi-bait construct into the Y1HGold reporter strain. Transformants were plated on SD/-Leu medium containing the AbA concentration established above. In the Empty pGADT7 control, no colony growth was observed on SD/-Leu/AbA plates for any of the bait constructs tested, confirming that the activation domain alone does not drive reporter expression. In the positive control (pGADT7-p53 co-transformed with p53-AbAi), robust colony growth was observed on AbA-containing medium. In the negative control (empty pGADT7 co-transformed with the respective pAbAi-bait construct) (Figure 3B), no colony growth was observed. All experiments were performed in the same batch of competent Y1HGold cells and identical incubation conditions for three independent transformations, with each performed on a separate day, and the same result was obtained in all three independent experiments.

### 4.7. Microscopy

For the subcellular localization of UMAG_06102 in maize cells, the full-length or N-terminal sequences were fused with GFP. The constructed plasmids were transfected with the freshly isolated protoplasts and the GFP signal was examined under a Leica SP8 confocal microscope (Leica Microsystems). Maize protoplast isolation and transient transfection were conducted according to the protocol as described previously [65]. To visualize the cellular location of UMAG_06102 in maize seedling leaves during *U. maydis* infection, tumor tissues collected from maize leaves inoculated with *U. maydis* for 12 days were incubated and frozen with tissue freeze medium (4% carboxymethyl cellulose sodium salt [*w/v*], Sigma Aldrich, catalog No. 419273) and sliced with a razor blade, then stained with WGA-AF488 (Invitrogen, catalog No. W11261), following the manufacturer’s instructions. The slide was visualized using a Leica SP8 confocal microscope (Leica Microsystems). The auto-fluorescence of the maize cell walls was excited at 405 nm and the emitted fluorescence was detected between 415 and 460 nm, while WGA-AF488-staining fluorescence was excited at 488 nm and emitted fluorescence detected between 495 and 530 nm; mCherry fluorescence was excited at 561nm and the emitted fluorescence detected between 580 and 630nm. The images were processed using LAS-AF software (lite 3.3.0) (Leica Microsystems).

## Data Availability

Sequence-based gene count datasets generated from this study have been deposited in the NCBI Gene Expression Omnibus (GEO) under the accession number GSE172071. Reference datasets used in this study are as follows: Steven J. Genome-Wide Transcription Factor Binding in Leaves from C3 and C4 Grasses. Maize seedling whole leaf. GSE97369. https://www.ncbi.nlm.nih.gov/geo/query/acc.cgi?acc=GSE97369 GSE97369_DGF_Zm_WL.bed (2019) (accessed on 10 August 2020). Rodgers-Melnick. Open chromatin reveals the functional maize genome. Maize seedling shoot. SRP064243. https://www.ncbi.nlm.nih.gov/sra?term=SRP064243 SRR2542698, SRR2542699, SRR2542700, SRR2542701 (2016) (accessed on 10 August 2020). William A. Widespread long-range cis-regulatory elements in the maize genome. Maize seedling leaf. GSE120304. https://www.ncbi.nlm.nih.gov/geo/query/acc.cgi?acc=GSE120304 GSM3398046_ATAC_B73_leaf.filtered_ACR.bed (2019) (accessed on 10 August 2020). Daniel Lanver. The Biotrophic Development of *Ustilago maydis* Studied by RNA-Seq Analysis. RNA-seq. GSE103876. https://www.ncbi.nlm.nih.gov/geo/query/acc.cgi?acc=GSE103876 GSE103876_Counts_Zm.txt (2018) (accessed on 10 August 2020). Alexandra Matei. How to make a tumour: cell type specific dissection of *Ustilago maydis*-induced tumour development in maize leaves. RNA-seq. SRP120945. https://www.ncbi.nlm.nih.gov/sra?term=SRP064243 SRR6202430, SRR6202431, SRR6202432, SRR6202433, SRR6202434, SRR6202435, SRR6202436, SRR6202437, SRR6202438, SRR6202439, SRR6202440, SRR6202441 (2018) (accessed on 10 August 2020).

## Supplementary Materials

The following supporting information can be downloaded at https://www.mdpi.com/article/10.3390/plants15203215/s1, Figure S1. (A) Dot-plot of cis-element GSEA analysis results of *F. graminearum*-induced maize genes. (B) Enrichment plot of RY-repeat in *F. graminearum*-infected RNA-seq dataset. Figure S2. Phylogenetic tree analysis of SWEET family in *Arabidopsis thaliana*, *Zea mays* and *Oryza sativa*. Figure S3. (A) Disease symptoms range from level 2 to level 5 upon inoculation with *U. maydis* wild-type (WT) and *Δ06102* for 8 days in CML326 and GEMS15. Plants with disease level 3 and level 4 in dashed border boxes were sampled for gene expression analysis. (B) Analysis of semi-quantitative PCR gene expression level; significance was tested with Student’s *t*-test and * indicates significant difference at *p* < 0.05 between WT and *Δ06102*-infected tumor samples. nd, not detected. Figure S4. (A) Disease symptom index distribution of maize line B73 upon inoculation for 8 days with *U. maydis* wild-type, *Δ06102* strain or *UMAG_06102::mCherry-HA* strain. The inoculation was performed three times independently, and disease symptom index distribution results were pooled for significance tests between each strain, performed with a Kruskal–Wallis test, where *** indicates *p* < 0.001. (B) Semi-quantitative PCR agarose-gel analysis of non-tumor (CT) and tumor (T) samples from seedlings with disease level 3 and level 4. nd, not detected. Figure S5. (A) Sequence alignment of three haplotypes of RY-repeat in *ZmSWEET11* promoter among maize lines. (B) Disease symptom index distribution of 83 maize lines grouped by three haplotypes of RY-repeat in *ZmSWEET11* promoter. Figure S6. (A) Disease symptom index of maize seedlings inoculated with *U. maydis* for 8 days. (B) The distribution of tumors on leaves in maize seedlings during *U. maydis* inoculation. (C) Disease symptoms of maize line seedlings resistant (CML326) and susceptible (GEMS15) to *U. maydis* after being infected for 8 days with *U. maydis*. (D) Disease symptom index distribution in resistant and susceptible maize line seedlings infected for 8 days with *U. maydis*; number of injected plants is labeled in the brackets and *** indicates significant different distribution in the Kruskal–Wallis test *p* < 0.001. Figure S7. Two compatible mating-type strains of *Ustilago maydis* (labelled as Um-c and Um-e) were used in this study. Mating-type loci were genotyped by PCR with locus-specific primers. (A) Primers UMfa1-F/UMfa1-R specifically amplify a 549-bp fragment from strain Um-c carrying the *a1* allele, whereas UMfa2-F/UMfa2-R specifically amplify a 637-bp fragment from strain Um-e carrying the *a2* allele. (B) Primers UMb1-F2/UMb1-R7 amplify an approximately 1296-bp fragment from the *b* locus across different genotypes, and the *b* locus genotype was determined by sequencing and alignment of the amplified products, which confirmed that strain Um-c harbors the *b3* allele and strain Um-e harbors the *b2* allele. Supplementary Table S1. RY-repeat gene list.xlsx: RY-repeat-containing genes listed in this study. Supplementary Table S2. Primer list.xlsx: primers used in this study.

## References

[1] Banuett F., Herskowitz I. (1989). Different alleles of *Ustilago maydis* are necessary for maintenance of filamentous growth but not for meiosis. Proc. Natl. Acad. Sci. USA.

[2] Skibbe D.S., Doehlemann G., Fernandes J., Walbot V. (2010). Maize tumors caused by *Ustilago maydis* require organ-specific genes in host and pathogen. Science.

[3] Lanver D., Tollot M., Schweizer G., Lo Presti L., Reissmann S., Ma L.-S., Schuster M., Tanaka S., Liang L., Ludwig N. (2017). *Ustilago maydis* effectors and their impact on virulence. Nat. Rev. Microbiol..

[4] Kretschmer M., Croll D., Kronstad J.W. (2017). Maize susceptibility to *Ustilago maydis* is influenced by genetic and chemical perturbation of carbohydrate allocation. Mol. Plant Pathol..

[5] Doehlemann G., Wahl R., Horst R.J., Voll L.M., Usadel B., Poree F., Stitt M., Pons-Kühnemann J., Sonnewald U., Kahmann R. (2008). Reprogramming a maize plant: Transcriptional and metabolic changes induced by the fungal biotroph *Ustilago maydis*. Plant J..

[6] Wahl R., Wippel K., Goos S., Kämper J., Sauer N. (2010). A Novel High-Affinity Sucrose Transporter Is Required for Virulence of the Plant Pathogen *Ustilago maydis*. PLoS Biol..

[7] Wittek A., Dreyer I., Al-Rasheid K.A.S., Sauer N., Hedrich R., Geiger D. (2017). The fungal UmSrt1 and maize ZmSUT1 sucrose transporters battle for plant sugar resources. J. Integr. Plant Biol..

[8] Schuler D., Wahl R., Wippel K., Vranes M., Münsterkötter M., Sauer N., Kämper J. (2015). Hxt1, a monosaccharide transporter and sensor required for virulence of the maize pathogen *Ustilago maydis*. New Phytol..

[9] Sosso D., van der Linde K., Bezrutczyk M., Schuler D., Schneider K., Kämper J., Walbot V. (2018). Sugar Partitioning between *Ustilago maydis* and Its Host *Zea may* L. during Infection. Plant Physiol..

[10] Eom J.-S., Chen L.-Q., Sosso D., Julius B.T., Lin I., Qu X.-Q., Braun D.M., Frommer W.B. (2015). SWEETs, transporters for intracellular and intercellular sugar translocation. Curr. Opin. Plant Biol..

[11] Yuan M., Zhao J., Huang R., Li X., Xiao J., Wang S. (2014). Rice MtN3/saliva/SWEET gene family: Evolution, expression profiling, and sugar transport. J. Integr. Plant Biol..

[12] Yang B., Sugio A., White F.F. (2006). Os8N3 is a host disease-susceptibility gene for bacterial blight of rice. Proc. Natl. Acad. Sci. USA.

[13] Liu Q., Yuan M., Zhou Y., Li X., Xiao J., Wang S. (2011). A paralog of the MtN3/saliva family recessively confers race-specific resistance to *Xanthomonas oryzae* in rice. Plant Cell Environ..

[14] Blanvillain-Baufumé S., Reschke M., Solé M., Auguy F., Doucoure H., Szurek B., Meynard D., Portefaix M., Cunnac S., Guiderdoni E. (2017). Targeted promoter editing for rice resistance to *Xanthomonas oryzae pv. oryzae* reveals differential activities for SWEET14-inducing TAL effectors. Plant Biotechnol. J..

[15] Cox K.L., Meng F., Wilkins K.E., Li F., Wang P., Booher N.J., Carpenter S.C.D., Chen L.-Q., Zheng H., Gao X. (2017). TAL effector driven induction of a SWEE gene confers susceptibility to bacterial blight of cotton. Nat. Commun..

[16] Walerowski P., Gündel A., Yahaya N., Truman W., Sobczak M., Olszak M., Rolfe S., Borisjuk L., Malinowski R. (2018). Clubroot Disease Stimulates Early Steps of Phloem Differentiation and Recruits SWEET Sucrose Transporters within Developing Galls. Plant Cell.

[17] Chong J., Piron M.-C., Meyer S., Merdinoglu D., Bertsch C., Mestre P. (2014). The SWEET family of sugar transporters in grapevine: VvSWEET4 is involved in the interaction with *Botrytis cinerea*. J. Exp. Bot..

[18] Schilling L., Matei A., Redkar A., Walbot V., Doehlemann G. (2014). Virulence of the maize smut *Ustilago maydis* is shaped by organ-specific effectors. Mol. Plant Pathol..

[19] Villajuana-Bonequi M., Matei A., Ernst C., Hallab A., Usadel B., Doehlemann G. (2019). Cell type specific transcriptional reprogramming of maize leaves during *Ustilago maydis* induced tumor formation. Sci. Rep..

[20] Schurack S., Depotter J., Gupta D., Thines M., Doehlemann G. (2021). Comparative transcriptome profiling identifies maize line specificity of fungal effectors in the maize-*Ustilago maydis* interaction. Plant J..

[21] Klemm S.L., Shipony Z., Greenleaf W.J. (2019). Chromatin accessibility and the regulatory epigenome. Nat. Rev. Genet..

[22] Rodgers-Melnick E., Vera D.L., Bass H.W., Buckler E.S. (2016). Open chromatin reveals the functional maize genome. Proc. Natl. Acad. Sci. USA.

[23] Zhao H., Zhang W., Chen L., Wang L., Marand A.P., Wu Y., Jiang J. (2018). Proliferation of Regulatory DNA Elements Derived from Transposable Elements in the Maize Genome. Plant Physiol..

[24] Ricci W.A., Lu Z., Ji L., Marand A.P., Ethridge C.L., Murphy N.G., Noshay J.M., Galli M., Mejía-Guerra M.K., Colomé-Tatché M. (2019). Widespread long-range cis-regulatory elements in the maize genome. Nat. Plants.

[25] Parvathaneni R.K., Bertolini E., Shamimuzzaman M., Vera D.L., Lung P.-Y., Rice B.R., Zhang J., Brown P.J., Lipka A.E., Bass H.W. (2020). The regulatory landscape of early maize inflorescence development. Genome Biol..

[26] Boch J., Scholze H., Schornack S., Landgraf A., Hahn S., Kay S., Lahaye T., Nickstadt A., Bonas U. (2009). Breaking the code of DNA binding specificity of TAL-type III effectors. Science.

[27] Kim S., Kim C.-Y., Park S.-Y., Kim K.-T., Jeon J., Chung H., Choi G., Kwon S., Choi J., Jeon J. (2020). Two nuclear effectors of the rice blast fungus modulate host immunity via transcriptional reprogramming. Nat. Commun..

[28] Horst R.J., Doehlemann G., Wahl R., Hofmann J., Schmiedl A., Kahmann R., Kämper J., Voll L.M. (2010). A model of *Ustilago maydis* leaf tumor metabolism. Plant Signal. Behav..

[29] Burgess S.J., Reyna-Llorens I., Stevenson S.R., Singh P., Jaeger K., Hibberd J.M. (2019). Genome Wide Transcription Factor Binding in Leaves from C3 and C4 Grasses. Plant Cell.

[30] Lanver D., Müller A.N., Happel P., Schweizer G., Haas F.B., Franitza M., Pellegrin C., Reissmann S., Altmüller J., Rensing S.A. (2018). The Biotrophic Development of Ustilago maydis Studied by RNA-Seq Analysis. Plant Cell.

[31] Matei A., Ernst C., Günl M., Thiele B., Altmüller J., Walbot V., Usadel B., Doehlemann G. (2018). How to make a tumour: Cell type specific dissection of *Ustilago maydis*-induced tumour development in maize leaves. New Phytol..

[32] Walbot V., Skibbe D.S. (2010). Maize host requirements for *Ustilago maydis* tumor induction. Sex. Plant Reprod..

[33] Suzuki M., Ketterling M.G., McCarty D.R. (2005). Quantitative Statistical Analysis of cis-Regulatory Sequences in ABA/VP1- and CBF/DREB1-Regulated Genes of Arabidopsis. Plant Physiol..

[34] Bäumlein H., Nagy I., Villarroel R., Inzé D., Wobus U. (1992). Cis-analysis of a seed protein gene promoter: The conservative RY repeat CATGCATG within the legumin box is essential for tissue-specific expression of a legumin gene. Plant J..

[35] Reidt W., Wohlfarth T., Ellerström M., Czihal A., Tewes A., Ezcurra I., Rask L., Bäumlein H. (2000). Gene regulation during late embryogenesis: The RY motif of maturation-specific gene promoters is a direct target of the FUS3 gene product. Plant J..

[36] Guerriero G., Martin N., Golovko A., Sundström J.F., Rask L., Ezcurra I. (2009). The RY/Sph element mediates transcriptional repression of maturation genes from late maturation to early seedling growth. New Phytol..

[37] Han Y., Zhang K., Yang J., Zhang N., Fang A., Zhang Y., Liu Y., Chen Z., Hsiang T., Sun W. (2015). Differential expression profiling of the early response to *Ustilaginoidea virens* between false smut resistant and susceptible rice varieties. BMC Genom..

[38] Tanaka S., Schweizer G., Rössel N., Fukada F., Thines M., Kahmann R. (2019). Neofunctionalization of the secreted Tin2 effector in the fungal pathogen *Ustilago maydis*. Nat. Microbiol..

[39] Chabouté M.E., Clément B., Sekine M., Philipps G., Chaubet-Gigot N. (2000). Cell cycle regulation of the tobacco ribonucleotide reductase small subunit gene is mediated by E2F-like elements. Plant Cell.

[40] Sozzani R., Maggio C., Varotto S., Canova S., Bergounioux C., Albani D., Cella R. (2006). Interplay between Arabidopsis activating factors E2Fb and E2Fa in cell cycle progression and development. Plant Physiol..

[41] Sánchez-Camargo V.A., Suárez-Espinoza C., Romero-Rodríguez S., Garza-Aguilar S.M., Stam M., García-Ramírez E., Lara-Núñez A., Vázquez-Ramos J.M. (2020). Maize E2F transcription factors. Expression, association to promoters of S-phase genes and interaction with the RBR1 protein in chromatin during seed germination. Plant Sci..

[42] Hudson M.E., Quail P.H. (2003). Identification of promoter motifs involved in the network of phytochrome A-regulated gene expression by combined analysis of genomic sequence and microarray data. Plant Physiol..

[43] Ökmen B., Kemmerich B., Hilbig D., Wemhöner R., Aschenbroich J., Perrar A., Huesgen P.F., Schipper K., Doehlemann G. (2018). Dual function of a secreted fungalysin metalloprotease in *Ustilago maydis*. New Phytol..

[44] Huang L., Ökmen B., Stolze S.C., Kastl M., Khan M., Hilbig D., Nakagami H., Djamei A., Doehlemann G. (2024). The fungal pathogen *Ustilago maydis* targets the maize corepressor RELK2 to modulate host transcription for tumorigenesis. New Phytol..

[45] Seebauer J.R., Singletary G.W., Krumpelman P.M., Ruffo M.L., Below F.E. (2010). Relationship of source and sink in determining kernel composition of maize. J. Exp. Bot..

[46] Julius B.T., Leach K.A., Tran T.M., Mertz R.A., Braun D.M. (2017). Sugar Transporters in Plants: New Insights and Discoveries. Plant Cell Physiol..

[47] Bezrutczyk M., Zöllner N.R., Kruse C.P.S., Hartwig T., Lautwein T., Köhrer K., Frommer W.B., Kim J.-Y. (2021). Evidence for phloem loading via the abaxial bundle sheath cells in maize leaves. Plant Cell.

[48] Chen L.-Q., Qu X.-Q., Hou B.-H., Sosso D., Osorio S., Fernie A.R., Frommer W.B. (2012). Sucrose Efflux Mediated by SWEET Proteins as a Key Step for Phloem Transport. Science.

[49] Chen L.-Q. (2014). SWEET sugar transporters for phloem transport and pathogen nutrition. New Phytol..

[50] Bezrutczyk M., Yang J., Eom J.-S., Prior M., Sosso D., Hartwig T., Szurek B., Oliva R., Vera-Cruz C., White F.F. (2018). Sugar flux and signaling in plant-microbe interactions. Plant J..

[51] Breia R., Conde A., Badim H., Fortes A.M., Gerós H., Granell A. (2021). Plant SWEETs: From sugar transport to plant--pathogen interaction and more unexpected physiological roles. Plant Physiol..

[52] An J., Zeng T., Ji C., de Graaf S., Zheng Z., Xiao T.T., Deng X., Xiao S., Bisseling T., Limpens E. (2019). A *Medicago truncatula* SWEET transporter implicated in arbuscule maintenance during arbuscular mycorrhizal symbiosis. New Phytol..

[53] Gupta P.K. (2020). SWEET Genes for Disease Resistance in Plants. Trends Genet..

[54] Li T., Huang S., Zhou J., Yang B. (2013). Designer TAL effectors induce disease susceptibility and resistance to *Xanthomonas oryzae pv. oryzae* in rice. Mol. Plant.

[55] Oliva R., Ji C., Atienza-Grande G., Huguet-Tapia J.C., Perez-Quintero A., Li T., Eom J.-S., Li C., Nguyen H., Liu B. (2019). Broad-spectrum resistance to bacterial blight in rice using genome editing. Nat. Biotechnol..

[56] Xu Z., Xu X., Gong Q., Li Z., Li Y., Wang S., Yang Y., Ma W., Liu L., Zhu B. (2019). Engineering Broad-Spectrum Bacterial Blight Resistance by Simultaneously Disrupting Variable TALE-Binding Elements of Multiple Susceptibility Genes in Rice. Mol. Plant.

[57] Redkar A., Doehlemann G. (2016). Ustilago maydis Virulence Assays in Maize. Bio-Protocol.

[58] Bösch K., Frantzeskakis L., Vraneš M., Kämper J., Schipper K., Göhre V. (2016). Genetic Manipulation of the Plant Pathogen *Ustilago maydis* to Study Fungal Biology and Plant Microbe Interactions. J. Vis. Exp..

[59] Pertea M., Pertea G.M., Antonescu C.M., Chang T.-C., Mendell J.T., Salzberg S.L. (2015). StringTie Enables Improved Reconstruction of a Transcriptome from RNA-Seq Reads. Nat. Biotechnol..

[60] Kim D., Langmead B., Salzberg S.L. (2015). HISAT: A Fast Spliced Aligner with Low Memory Requirements. Nat. Methods.

[61] Love M.I., Huber W., Anders S. (2014). Moderated Estimation of Fold Change and Dispersion for RNA-Seq Data with DESeq2. Genome Biol..

[62] Grant C.E., Bailey T.L., Noble W.S. (2011). FIMO: Scanning for Occurrences of a given Motif. Bioinformatics.

[63] Higo K., Ugawa Y., Iwamoto M., Korenaga T. (1999). Plant Cis-Acting Regulatory DNA Elements (PLACE) Database: 1999. Nucleic Acids Res..

[64] Subramanian A., Tamayo P., Mootha V.K., Mukherjee S., Ebert B.L., Gillette M.A., Paulovich A., Pomeroy S.L., Golub T.R., Lander E.S. (2005). Gene Set Enrichment Analysis: A Knowledge-Based Approach for Interpreting Genome-Wide Expression Profiles. Proc. Natl. Acad. Sci. USA.

[65] Gao X.Q., Wheeler T., Li Z.H., Kenerley C.M., He P., Shan L.B. (2011). Silencing GhNDR1 and GhMKK2 compromises cotton resistance to Verticillium wilt. Plant J..

