# Supplementary material for: Enrichment of the Conserved RY-Repeat Cis-Element in Maize upon Ustilago maydis Infection and Its Interaction with the Candidate Secreted Protein UMAG_06102
Source: Plants (Basel). 2026 Oct 20;15(20):3215. doi: 10.3390/plants15203215 (PMC13709229; doi:10.3390/plants15203215)
Supplement: Supplementary file 1 [file plants-15-03215-s001.zip › Supplemental Figures-R3-20261013.pdf]

Supplemental Figures

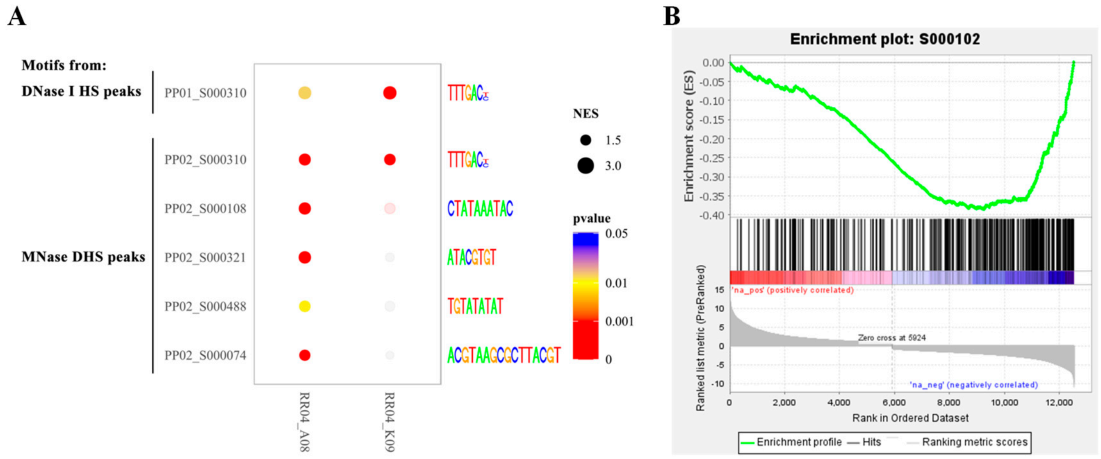

Figure S1. (A) Dot-plot of cis-element GSEA analysis results of *F. graminearum*-induced maize genes. (B) Enrichment plot of RY-repeat in *F. graminearum*-infected RNA-seq dataset.

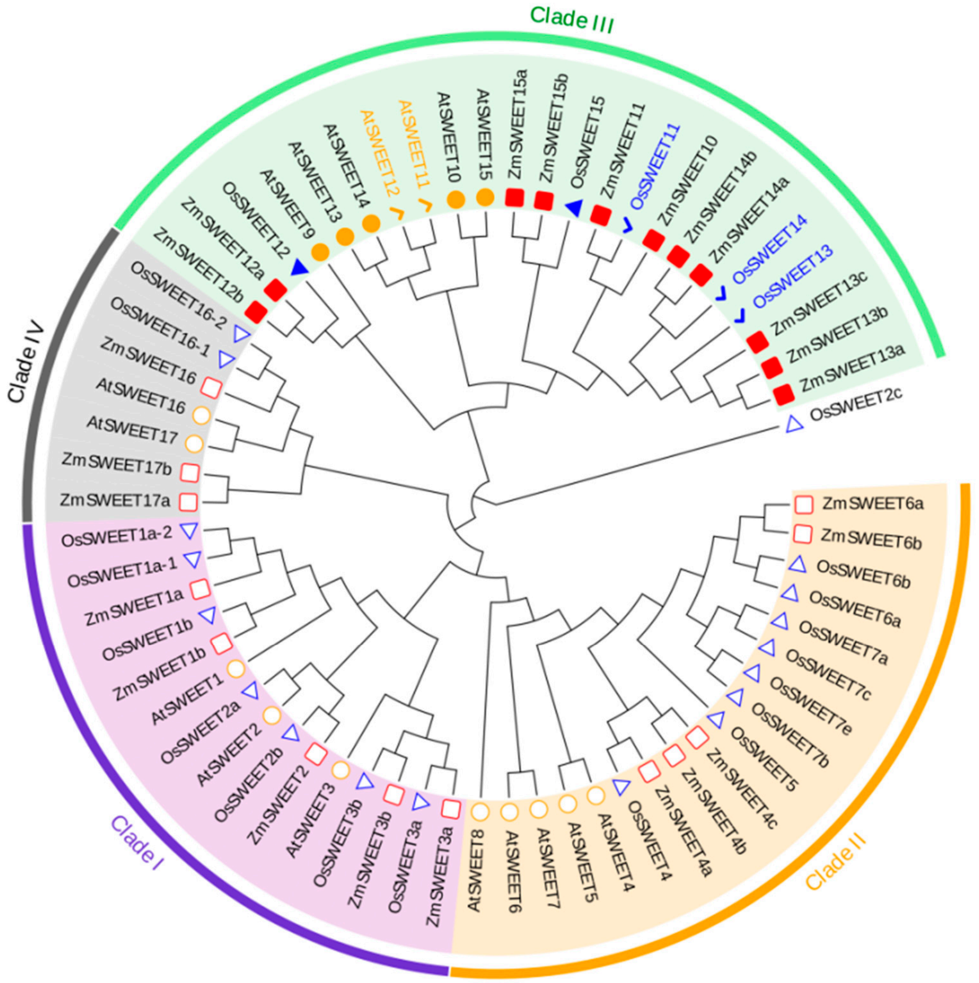

Figure S2. Phylogenetic tree analysis of SWEET family in *Arabidopsis thaliana*, *Zea mays* and *Oryza sativa*.

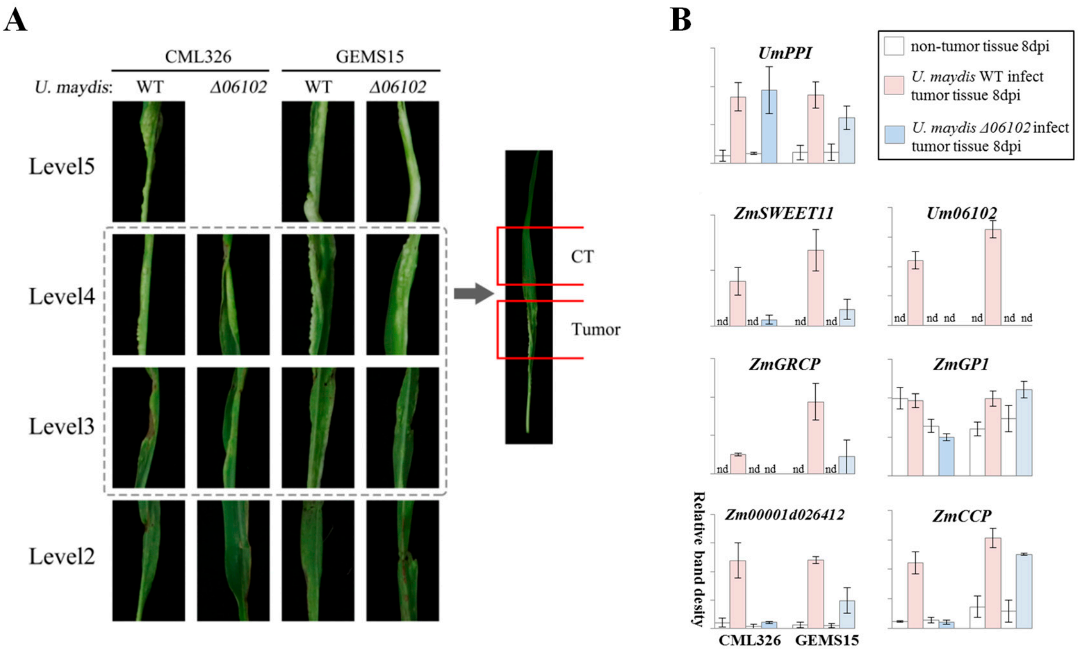

Figure S3. (A) Disease symptoms range from level 2 to level 5 upon inoculation with U. maydis wild-type (WT) and *Δ06102* for 8 days in CML326 and GEMS15. Plants with disease level 3 and level 4 in dashed border boxes were sampled for gene expression analysis. (B) Analysis of semi-quantitative PCR gene expression level; significance was tested with Student's *t*-test and * indicates significant difference at $p < 0.05$ between WT and *Δ06102*-infected tumor samples. nd, not detected.

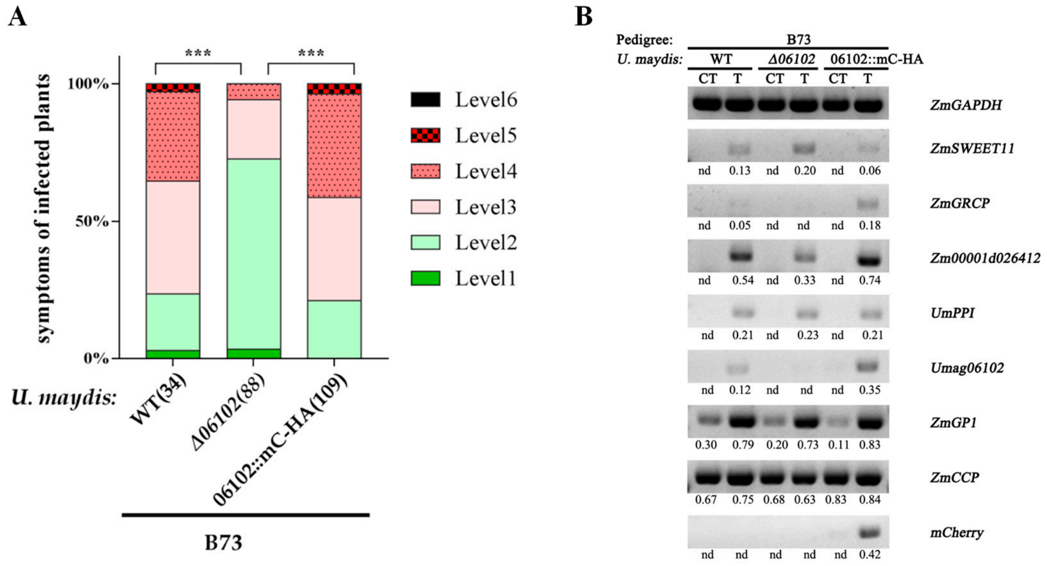

Figure S4. (A) Disease symptom index distribution of maize line B73 upon inoculation for 8 days with *U. maydis* wild-type, *Δ06102* strain or *UMAG_06102::mCherry-HA* strain. The inoculation was performed three times independently, and disease symptom index distribution results were pooled for significance tests between each strain, performed with a Kruskal–Wallis test, where *** indicates $p < 0.001$. (B) Semi-quantitative PCR agarose-gel analysis of non-tumor (CT) and tumor (T) samples from seedlings with disease level 3 and level 4. nd, not detected.

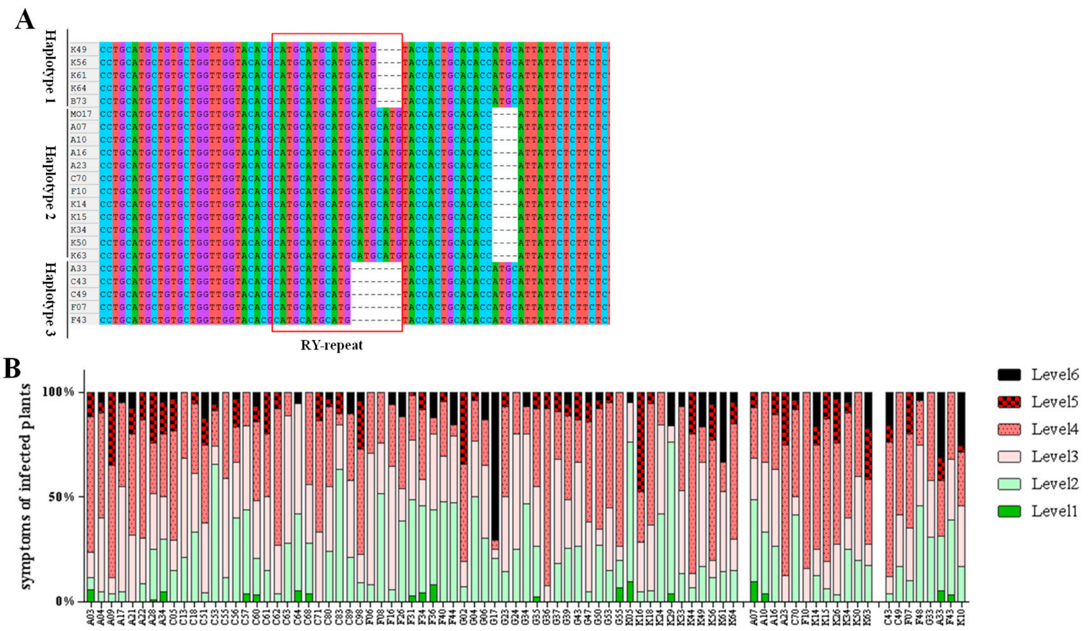

Figure S5. (A) Sequence alignment of three haplotypes of RY-repeat in *ZmSWEET11* promoter among maize lines. (B) Disease symptom index distribution of 83 maize lines grouped by three haplotypes of RY-repeat in *ZmSWEET11* promoter.

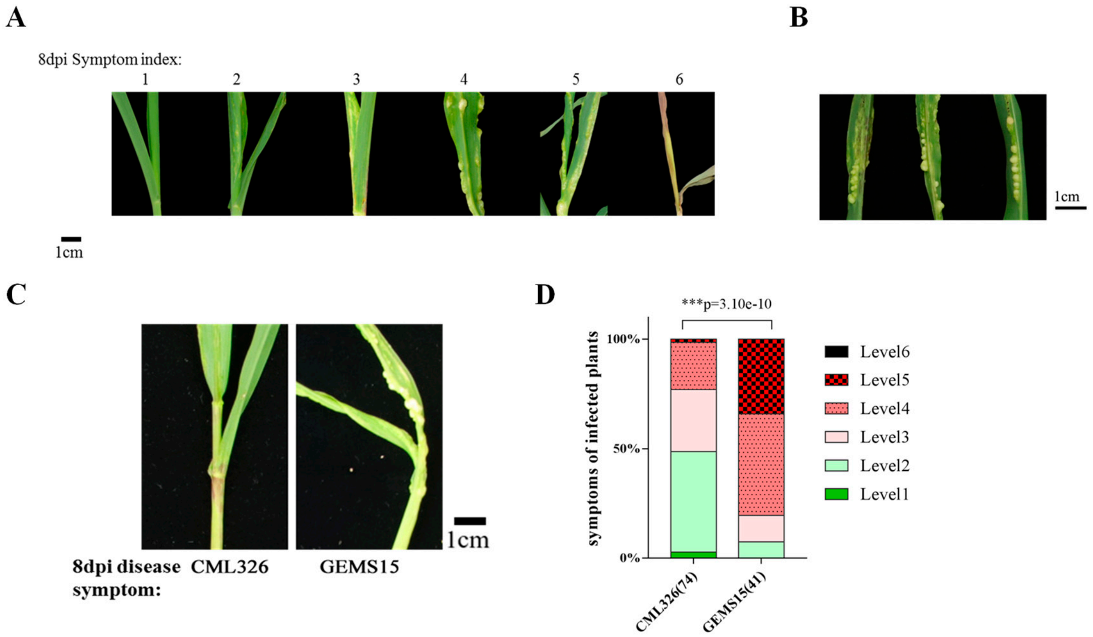

Figure S6. (A) Disease symptom index of maize seedlings inoculated with *U. maydis* for 8 days. (B) The distribution of tumors on leaves in maize seedlings during *U. maydis* inoculation. (C) Disease symptoms of maize line seedlings resistant (CML326) and susceptible (GEMS15) to *U. maydis* after being infected for 8 days with *U. maydis*. (D) Disease symptom index distribution in resistant and susceptible maize line seedlings infected for 8 days with *U. maydis*; number of injected plants is labeled in the brackets and *** indicates significant different distribution in the Kruskal–Wallis test $p < 0.001$.

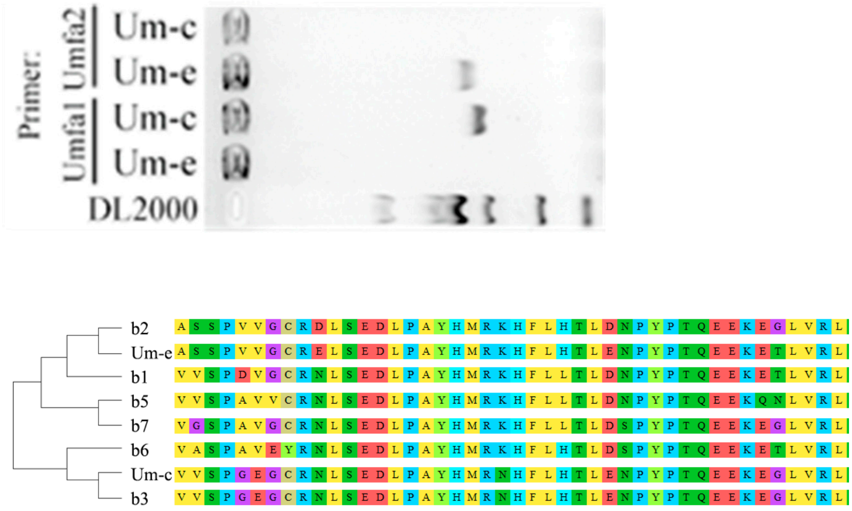

Figure S7. Two compatible mating-type strains of *Ustilago maydis* (labelled as Um-c and Um-e) were used in this study. Mating-type loci were genotyped by PCR with locus-specific primers. (A) Primers UMfa1-F/UMfa1-R specifically amplify a 549-bp fragment from strain Um-c carrying the *a1* allele, whereas UMfa2-F/UMfa2-R specifically amplify a 637-bp fragment from strain Um-e carrying the *a2* allele. (B) Primers UMb1-F2/UMb1-R7 amplify an approximately 1296-bp fragment from the *b* locus across different genotypes, and the *b* locus genotype was determined by sequencing and alignment of the amplified products, which confirmed that strain Um-c harbors the *b3* allele and strain Um-e harbors the *b2* allele.
